# Schisandrin B Attenuates Renal Fibrotic Remodeling in Association with Restoration of a PPARα-Related Tubular Fatty-Acid Oxidation Program

**DOI:** 10.3390/biomedicines14061351

**Published:** 2026-06-15

**Authors:** Yun Deng, Changhong Xu, Jiaxuan Ma, Rui Yan, Yalong Zhang, Hao Wang, Kangyu Wang, Jiangwei Man, Li Yang

**Affiliations:** Department of Urology, Gansu Province Clinical Research Center for Urinary System Disease, The Second Hospital & Clinical Medical School, Lanzhou University, No. 82 Cuiyingmen, Lanzhou 730030, China; dengy240072@163.com (Y.D.);

**Keywords:** Schisandrin B, PPARα, fatty acid β-oxidation, renal ischemia–reperfusion injury, tubulointerstitial fibrosis

## Abstract

**Background:** Renal ischemia–reperfusion injury (RIRI) is a major cause of acute kidney injury (AKI) and contributes to delayed graft function and progression toward chronic kidney disease. In addition to oxidative stress and inflammation, RIRI induces profound metabolic derangements, particularly suppression of tubular fatty-acid β-oxidation (FAO), leading to energetic stress, lipid accumulation, and maladaptive repair. Peroxisome proliferator–activated receptor-α (PPARα) is a key regulator of tubular FAO, but whether Schisandrin B (Sch B) mitigates RIRI through restoration of a PPARα-associated metabolic program remains unclear. **Objective:** To determine whether Sch B alleviates RIRI in association with restoration of tubular FAO and attenuation of lipid accumulation and fibrotic remodeling. **Methods:** A unilateral murine renal I/R model and an HK-2 hypoxia/reoxygenation (H/R) model were used. Mice received Sch B (20 or 40 mg/kg/day) before I/R, and a subset was co-treated with the PPARα antagonist GW6471. Renal function, tubular injury, fibrosis, lipid accumulation, and FAO-related proteins were assessed by serum biochemistry, histopathology, Oil Red O staining, transmission electron microscopy, immunohistochemistry, immunofluorescence, and Western blotting. Bulk RNA-seq and public single-cell RNA-seq datasets were integrated to characterize metabolic pathway remodeling and cell-type-associated PPARα changes. Molecular docking and molecular dynamics simulations were performed to explore the potential interaction between Sch B and PPARα. **Results:** Sch B significantly improved renal function, reduced tubular injury, and attenuated interstitial collagen deposition after I/R. Sch B also reduced lipid droplet accumulation, preserved mitochondrial ultrastructure, and restored the expression of FAO-related proteins, including CPT1A, CPT2, and ACADM. In vivo and in vitro, Sch B decreased α-SMA, COL1A1, and vimentin expression, indicating attenuation of EMT-associated/profibrotic remodeling. Integrated transcriptomic analyses supported marked metabolic reprogramming after I/R, with enrichment of FAO- and PPAR-related pathways and reduced PPARα expression predominantly in tubular compartments. Sch B was associated with restoration of tubular PPARα expression, while docking and molecular dynamics analyses supported a plausible Sch B–PPARα interaction in silico. GW6471 blunted the beneficial effects of Sch B on fibrosis-related and FAO-related readouts. **Conclusions:** Sch B alleviates RIRI and limits subsequent fibrotic remodeling in association with restoration of a PPARα-related tubular FAO program, reduced lipid accumulation, and preservation of tubular metabolic homeostasis. These findings identify metabolic reprogramming as an important component of Sch B-mediated renoprotection, although the precise mode by which Sch B regulates PPARα requires further investigation.

## 1. Introduction

RIRI is a major cause of AKI and occurs frequently in clinical settings such as kidney transplantation and major surgery. The pathological process comprises an initial ischemic insult followed by reperfusion-induced secondary damage, during which bursts of oxidative stress, inflammation, and regulated forms of cell death exacerbate renal injury [1,2]. Beyond the acute phase, severe RIRI can trigger maladaptive repair, leading to progressive tubulointerstitial fibrosis and chronic kidney dysfunction [3]. Clinically, RIRI-related fibrosis is closely associated with delayed graft function (DGF) and long-term graft loss [4]. Despite its clinical importance, no effective targeted therapy exists; management remains largely supportive, underscoring the need for new renoprotective strategies.

A central nexus in RIRI pathophysiology is the disruption of mitochondrial energy metabolism in renal tubular epithelial cells. Under physiological conditions, proximal tubules rely heavily on oxidative metabolism, with mitochondrial fatty acid oxidation (FAO) serving as the primary energy source to meet their substantial ATP demand [5]. FAO efficiency is critical for sustaining ion transport and other energy-intensive processes in cortical nephrons. PPARα orchestrates the expression of genes involved in fatty-acid uptake, β-oxidation enzymes, and lipid catabolism, acting as a central regulator of renal lipid metabolic homeostasis [6]. I/R perturbs this axis, impairing mitochondrial function and PPARα signaling, compromising fatty-acid utilization and precipitating an energetic crisis that threatens tubular cell survival.

Accumulating evidence indicates that FAO dysfunction is not merely a consequence of AKI but a driver of injury propagation and fibrosis [7]. Suppression of PPARα signaling—whether due to ischemia, aging, or genetic defects—leads to lipid accumulation, ATP depletion, and cellular stress, which in turn promote tubular cell death and dedifferentiation, activate profibrotic pathways, and impede normal repair [8]. In experimental models, loss of PPARα or pharmacological blockade of FAO exacerbates post-injury interstitial fibrosis, whereas preserving or enhancing PPARα activity ameliorates AKI severity and restrains subsequent fibrosis by maintaining energetic sufficiency and preventing lipotoxicity [7,9,10]. Thus, the PPARα–FAO axis is an attractive therapeutic target to disrupt the transition from acute injury to chronic fibrotic remodeling. In this context, persistent lipid metabolic derangement and lipotoxic stress are increasingly recognized as important contributors to maladaptive repair and AKI-to-CKD transition after ischemic injury [3,11,12].

Natural product-derived compounds are of interest for multitarget organ protection. Sch B, a bioactive lignan from *Schisandra chinensis*, has long been used for hepatoprotection and exhibits broad cytoprotective effects across organs. In diabetic cardiomyopathy and myocardial ischemia, Sch B attenuates damage through antioxidant and anti-apoptotic actions [13,14]. In acute liver injury and lipopolysaccharide-induced acute lung injury, it reduces inflammatory mediator release and oxidative stress, limits apoptosis, and preserves tissue architecture [15,16]. Mechanistic studies show that Sch B stabilizes mitochondrial function under stress and can activate PI3K/Akt survival signaling [17]. Our prior work showed that Sch B engages AKT1 and modulates mitochondrial dynamics to improve outcomes in RIRI [18]; however, its impact on chronic sequelae after AKI remains unclear.

Therefore, we investigated whether Sch B alleviates renal ischemia–reperfusion injury and subsequent fibrotic remodeling in association with restoration of tubular FAO. Using murine renal I/R and HK-2 hypoxia/reoxygenation models, together with transcriptomic analyses, pharmacological inhibition, and in silico simulations, we explored the potential involvement of a PPARα-related metabolic program in Sch B-mediated renoprotection. We hypothesized that Sch B may mitigate post-ischemic tubular injury and fibrotic remodeling by preserving tubular lipid metabolic homeostasis, while recognizing that direct receptor activation requires further validation.

## 2. Materials and Methods

### 2.1. Reagents and Antibodies

Schisandrin B (Sch B, S3600) and the PPARα antagonist GW6471 (S2798) were purchased from Selleck (Shanghai, China). Primary antibodies against PPARα (66826-1-Ig) and HRP-conjugated anti-mouse IgG (SA00001-1) were obtained from Proteintech (Wuhan, China). Antibodies against α-smooth muscle actin (α-SMA, SY02-64), collagen I (COL1A1, ST58-04), vimentin (SC60-05), carnitine palmitoyltransferase 1A (CPT1A, PSH04-00), carnitine palmitoyltransferase 2 (CPT2, SN06-70), and medium-chain acyl-CoA dehydrogenase (ACADM, JE48-09) were from Habio (Hangzhou, China). HE, PAS, Masson, Sirius Red, Oil Red O staining kits, and DAPI were purchased from Solarbio (Beijing, China). The IHC detection kit was obtained from Fuzhou Maixin Biotech (Fuzhou, China). Human proximal tubular epithelial HK-2 cells were purchased from the Cell Bank of Type Culture Collection of the Chinese Academy of Sciences (Shanghai, China).

### 2.2. Bulk RNA Sequencing and Enrichment Analysis

Total RNA was extracted from frozen kidney cortex tissues (I/R and I/R+Sch B groups) using TRIzol reagent. RNA concentration and integrity were assessed by spectrophotometry (Thermo Fisher Scientific, Waltham, MA, USA) and Bioanalyzer (Agilent Technologies, Santa Clara, CA, USA), and only samples with adequate purity and RNA integrity were used for library preparation. RNA sequencing libraries were prepared using a standard Illumina kit (Illumina, San Diego, CA, USA) and sequenced on an Illumina platform (Illumina, San Diego, CA, USA) in paired-end mode. Raw reads were quality-controlled and trimmed, then aligned to the mouse reference genome using HISAT2. Gene counts were obtained and normalized, and differentially expressed genes (DEGs) between groups were identified using DESeq2 with |log_2_ fold change| > 1 and adjusted *p* < 0.05 (Benjamini–Hochberg correction). Significantly regulated genes were subjected to Gene Ontology (GO) and Kyoto Encyclopedia of Genes and Genomes (KEGG) pathway enrichment analyses using the clusterProfiler package (version 4.8.3) in R (version 4.3.2). Enriched pathways related to lipid metabolism, fatty acid oxidation (FAO), and fibrosis were visualized using bar plots and bubble plots.

### 2.3. Single-Cell RNA-seq Analysis and Pseudotime Inference

Publicly available single-cell RNA-seq datasets of mouse kidneys subjected to sham/control or ischemia–reperfusion (I/R) were analyzed using Seurat (v4) and Monocle3 in R (version 4.3.2). For each 10× Genomics sample, UMI count matrices were imported with Read10X/CreateSeuratObject and merged into a single Seurat object. Low-quality cells with fewer than 50 detected genes or a mitochondrial gene percentage greater than 15% were excluded. Data were normalized using the “LogNormalize” method (scale factor 10,000), and 1500 highly variable genes were identified. Principal component analysis was performed, and the first 20 principal components were used as input for Harmony integration to correct batch effects across samples using sample ID as the batch covariate. Harmony embeddings (dims 1–20) were used to construct a shared nearest-neighbor graph and perform clustering with FindNeighbors and FindClusters; a resolution of 0.6 was selected for downstream analyses. Two-dimensional visualization was generated by UMAP based on the Harmony space.

Clusters were annotated into major kidney cell types, including proximal tubule, thick ascending limb, distal convoluted tubule, collecting duct, endothelial cells, fibroblasts, podocytes, and immune subsets, using canonical marker genes and visualized by dot plots and UMAP. A “Condition” variable (Control vs. IR) was derived from sample information, and cells with ambiguous labels were excluded. Cell type-specific differential expression between Control and IR was assessed with Seurat (FindMarkers), and significantly changed genes (adjusted *p* < 0.05 with biologically meaningful fold change) were subjected to GO/KEGG enrichment with a focus on FAO- and Ppara-related pathways. For trajectory inference, Monocle3 was applied to tubular epithelial cells; UMAP coordinates from Seurat were used as the low-dimensional embedding, and control proximal tubule cells were set as the root state. Pseudotime was estimated to model injury–repair trajectories, and the expression dynamics of FAO- and Ppara-related genes were examined along pseudotime. The single-cell RNA-seq datasets used in this study are publicly available in the NCBI Gene Expression Omnibus (GEO) under accession numbers GSE244330 and GSE249928.

### 2.4. Molecular Docking

The two-dimensional structure of Sch B was retrieved from PubChem and converted into an energy-minimized three-dimensional conformation using ChemBio3D 14.0. The crystal structure of the PPARα ligand-binding domain was downloaded from the RCSB Protein Data Bank and prepared by removing water molecules and co-crystallized ligands and by adding hydrogens using AutoDockTools 1.5.6 and PyMOL 2.5.4. The docking grid box was centered on the canonical ligand-binding pocket. Docking between Sch B and PPARα was performed using AutoDock Vina 1.1.2 with an exhaustiveness value of 128. Binding poses were ranked according to predicted binding affinity, and the lowest-energy, geometrically reasonable pose was selected for visualization and subsequent molecular dynamics simulations.

### 2.5. Molecular Dynamics (MD) Simulations

Molecular dynamics (MD) simulations of the Sch B–PPARα complex were carried out using GROMACS 2021.5. The protein was described using an Amber-compatible force field, and Sch B parameters were generated with AmberTools 20 with the general AMBER force field (GAFF), with RESP charges calculated using Gaussian 16. The complex was solvated in a TIP3P water box with counter-ions added to neutralize the system. After energy minimization, the system underwent NVT and NPT equilibration (100 ps each, 300 K, 1 bar), followed by a 100 ns production run with a 2-fs time step. Trajectories were analyzed for root-mean-square deviation (RMSD), root-mean-square fluctuation (RMSF), radius of gyration (Rg), solvent-accessible surface area (SASA), and protein–ligand hydrogen bonds. Binding free energies and per-residue contributions were estimated using an MM/GBSA approach, and free-energy landscapes were constructed to identify dominant low-energy conformations of the complex.

### 2.6. Cell Culture, Hypoxia/Reoxygenation and Sch B Treatment

Human proximal tubular epithelial HK-2 cells were cultured in DMEM/F-12 medium supplemented with 10% fetal bovine serum, 100 U/mL penicillin, and 100 μg/mL streptomycin at 37 °C in a humidified incubator containing 5% CO_2_. To mimic renal ischemia–reperfusion injury in vitro, HK-2 cells were subjected to hypoxia/reoxygenation (H/R). Briefly, cells were incubated in serum-free, low-glucose medium under hypoxic conditions (1% O_2_, 5% CO_2_, and 94% N_2_) for 12 h and then returned to normoxic conditions (95% air and 5% CO_2_) in complete DMEM/F-12 containing 10% fetal bovine serum for at least 2 h. For Sch B pretreatment, cells were incubated with Sch B (20 or 40 μM; designated S20 and S40, respectively) for 12 h before hypoxia and maintained at the same concentrations during the H/R procedure. Control cells (N and N+Sch B) were maintained under normoxic conditions with vehicle or 40 μM Sch B. Unless otherwise stated, in vitro quantitative experiments were performed in at least three independent biological replicates.

### 2.7. Transmission Electron Microscopy (TEM)

To assess mitochondrial ultrastructure and intracellular lipid accumulation, small cortical blocks from the left kidney were immediately fixed in 2.5–3% glutaraldehyde in 0.1 mol/L phosphate buffer at 4 °C, post-fixed in 1% osmium tetroxide, dehydrated through graded ethanol, and embedded in epoxy resin. Ultrathin sections (60–90 nm) were cut, stained with uranyl acetate and lead citrate, and examined using a JEM-1400FLASH transmission electron microscope (JEOL, Tokyo, Japan). Representative proximal tubular epithelial cells were imaged for mitochondrial morphology and lipid droplets.

### 2.8. Immunofluorescence (IF)

For immunofluorescence, HK-2 cells were seeded onto poly-L-lysine-coated coverslips and treated as described above (N, N+Sch B 40 μM, HR, HR+S20, and HR+S40). Cells were fixed with 4% paraformaldehyde for 15–20 min, permeabilized with 0.3% Triton X-100 (Solarbio, Beijing, China), and blocked with 5% bovine serum albumin. Primary antibodies against α-SMA, COL1A1, vimentin, or PPARα were incubated overnight at 4 °C, followed by appropriate fluorophore-conjugated secondary antibodies for 1 h at room temperature in the dark. Nuclei were counterstained with DAPI. Images were acquired using a laser scanning confocal microscope with a 63× oil-immersion objective (overall magnification approximately 630×). Mean fluorescence intensity was quantified in at least five randomly selected fields per coverslip using ImageJ 1.53t software.

### 2.9. Oil Red O Staining

After H/R and Sch B treatment (N, HR, HR+S20, and HR+S40), HK-2 cells grown in 12-well plates were fixed with 4% paraformaldehyde for 30 min at room temperature, rinsed with PBS, equilibrated in 60% isopropanol for 5 min, and stained with freshly prepared Oil Red O working solution for 30 min. Nuclei were counterstained with hematoxylin for 1 min. Images were captured by light microscopy, and intracellular lipid accumulation was quantified as the percentage of Oil Red O-positive area per field using ImageJ.

### 2.10. Ethical Compliance

All procedures were approved by the Animal Ethics Committee of the Second Hospital of Lanzhou University (Approval No. D2025-461). Animal care and use complied with the Guidelines for the Ethical Review of Laboratory Animal Welfare issued by the Ministry of Science and Technology of the People’s Republic of China. This study was conducted and reported in accordance with the ARRIVE guidelines.

### 2.11. Animal Experiments and Renal Ischemia–Reperfusion Model

#### 2.11.1. Sch B Dose–Response Experiment

Male C57BL/6 mice (8 weeks old, 20–25 g) were obtained from the Lanzhou Veterinary Research Institute and housed under specific-pathogen-free conditions with free access to food and water under a 12 h light/dark cycle. Mice were acclimatized for at least 1 week before experimentation. Animals were randomly assigned to five groups (*n* = 5 mice/group unless otherwise stated): Sham, Sham+Sch B (20 mg/kg/day), IR, IR+S20 (Sch B 20 mg/kg/day), and IR+S40 (Sch B 40 mg/kg/day). Sch B was dissolved in corn oil and administered by oral gavage once daily for 7 days before surgery and continued until sacrifice. Sham and IR mice received the same volume of vehicle. Unilateral renal ischemia–reperfusion was induced under anesthesia. Briefly, a flank incision was made to expose the left kidney, and the left renal pedicle was clamped with a non-traumatic microvascular clip for 45 min until the kidney turned pale. The clip was then removed to allow reperfusion, and the incision was closed. Sham and Sham+Sch B mice underwent the same procedure without clamping. Mice were sacrificed at 3, 7, and 14 days after I/R. Blood and kidneys were collected for renal function tests, histology, immunohistochemistry, immunofluorescence, transmission electron microscopy, Western blotting, and other molecular analyses.

#### 2.11.2. PPARα Inhibition Experiment with GW6471

To evaluate the dependence of Sch B on PPARα, an independent cohort of mice was randomized into four groups: IR, IR+Sch B, IR+GW6471, and IR+GW6471+Sch B. The I/R procedure was identical to that described above. Sch B (40 mg/kg/day) was administered by gavage for 7 days before I/R and maintained during the observation period. GW6471 was dissolved in a small volume of DMSO and diluted in corn oil, then injected intraperitoneally at 20 mg/kg once daily, starting 30 min before Sch B administration. Mice were sacrificed at 3, 7, and 14 days after I/R for evaluation of fibrosis- and fatty acid oxidation-related markers.

### 2.12. Anesthesia and Euthanasia

Mice were anesthetized with inhaled isoflurane (induction 3–4%, maintenance 1.5–2% in oxygen). Adequate anesthesia was confirmed by loss of the pedal withdrawal reflex. At the end of the experiment, mice were euthanized in accordance with the AVMA Guidelines using CO_2_ with a gradual-fill displacement rate of 30–70% of the chamber volume per minute (without pre-filling the chamber), followed by a secondary physical method to ensure death.

### 2.13. Western Blotting

Total protein was extracted from renal cortex using NP-40 lysis buffer supplemented with protease inhibitors. Lysates were centrifuged at 12,000× *g* for 15 min at 4 °C, and the supernatants were mixed with 4× loading buffer and boiled for 10 min. Equal amounts of protein (30 μg per lane) were separated on 10% SDS–PAGE gels and transferred onto PVDF membranes. After blocking with 5% non-fat milk in TBS-T for 2 h at room temperature, membranes were incubated overnight at 4 °C with primary antibodies against α-SMA and GAPDH. After washing, membranes were incubated with HRP-conjugated secondary antibodies for 1 h at room temperature. Bands were visualized by enhanced chemiluminescence and quantified using ImageJ; α-SMA expression was normalized to GAPDH. The specific GAPDH band used for normalization was selected consistently according to molecular weight and band specificity. For Western blotting, renal cortical protein lysates from independent animals were used as biological replicates. Each lane represented one individual mouse sample rather than repeated loading or repeated measurement of the same lysate. Three independent biological replicates were included for each group. The original uncropped western blot images and corresponding lane identities are provided in Supplementary Figures S1–S43 and/or the Source Data file. For densitometric analysis of normalized western blot data, the α-SMA/GAPDH value from each individual biological replicate was retained and then normalized to the mean value of the designated reference group at the same time point. Therefore, the reference group had a mean value of 1 after normalization, while replicate-level variability was preserved for SEM calculation and statistical analysis.

### 2.14. Renal Function

Blood samples were centrifuged at 3000 rpm for 15 min to obtain serum. Serum urea and creatinine levels were measured using commercial colorimetric kits according to the manufacturers’ protocols.

### 2.15. Histopathology and Fibrosis Scoring

Kidneys were fixed in 4% paraformaldehyde, dehydrated, embedded in paraffin, and sectioned at 3–4 μm. Sections were stained with HE and PAS to evaluate tubular injury, and with Masson trichrome and Sirius Red to assess interstitial collagen deposition. Tubular injury was semi-quantitatively scored in corticomedullary fields (×200) by blinded observers using a 0–5 scale based on tubular dilation, brush-border loss, cast formation, and epithelial cell detachment (0, none; 1, <10%; 2, 10–25%; 3, 26–50%; 4, 51–75%; 5, >75% of tubules affected). For each kidney, multiple non-overlapping random fields were analyzed and averaged to generate one biological value per animal. Interstitial fibrosis was quantified as the percentage of Masson- or Sirius Red-positive area in the cortex using ImageJ.

### 2.16. Immunohistochemistry (IHC)

Paraffin-embedded sections were deparaffinized in xylene, rehydrated through graded ethanol, and subjected to antigen retrieval in citrate buffer (pH 6.0). After blocking endogenous peroxidase with 3% H_2_O_2_ and non-specific binding with 5% bovine serum albumin, sections were incubated overnight at 4 °C with primary antibodies against α-SMA, COL1A1, vimentin, CPT1A, CPT2, ACADM, or PPARα. After washing, sections were incubated with HRP-conjugated secondary antibodies and visualized using DAB. Nuclei were counterstained with hematoxylin. Images were acquired under identical exposure settings with a light microscope. The percentage of positive staining area in the renal cortex was quantified in at least five randomly selected fields per section using ImageJ by an observer blinded to group allocation.

### 2.17. Sample Size, Randomization, Blinding, and Reproducibility

The sample size was determined based on experimental feasibility, prior experience with renal ischemia–reperfusion models, and the use of multiple complementary readouts across different time points rather than a formal a priori power calculation. Each mouse was treated as one independent biological replicate. Animals were randomly assigned to experimental groups before surgery. Histological scoring and immunohistochemical quantification were performed using multiple non-overlapping fields per kidney by observers blinded to group allocation. For in vitro assays, experiments were performed using at least three independent biological replicates unless otherwise stated. Western blotting was performed using renal cortical lysates from independent animals, and each lane represented one individual mouse sample. Uncropped blots and lane identities were provided in the Source Data file.

### 2.18. Statistics Analysis

Data are presented as mean ± SEM. Each animal was treated as one biological replicate for in vivo experiments. For in vitro assays, the number of independent biological replicates is indicated in the corresponding figure legends and was typically at least three independent experiments unless otherwise stated. For normalized Western blot quantification, statistical analyses were performed using replicate-level normalized values rather than group means alone. Reference groups were normalized to a mean value of 1, but SEM values were calculated from the individual biological replicates within each reference group. Statistical analyses were performed using GraphPad Prism 10.0. For comparisons among multiple groups, one-way ANOVA followed by Dunnett’s multiple-comparison test was used when data approximated a normal distribution and variances were comparable. Data distributions were examined in GraphPad Prism before parametric testing. A two-sided *p* < 0.05 was considered statistically significant. For injury-related outcomes, Sham animals were considered the physiological baseline. Therefore, the primary statistical interpretation focused on injury induction, including IR versus Sham, and treatment-related reversal after injury, including IR+Sch B versus IR. Minor numerical differences between Sham and Sham+Sch B groups were not interpreted as biological improvement beyond baseline, and Sham versus Sham+Sch B comparisons were not used to support any renoprotective conclusion.

### 2.19. Source Data Availability

Uncropped Western blot images and lane identities are provided in the Supplementary Materials or Source Data file. Each western blot lane corresponds to one individual mouse sample. Other numerical data supporting the quantitative analyses are available from the corresponding author upon reasonable request. Public single-cell RNA-seq datasets analyzed in this study are available in GEO under accession numbers GSE244330 and GSE249928.

**Figure 1:**
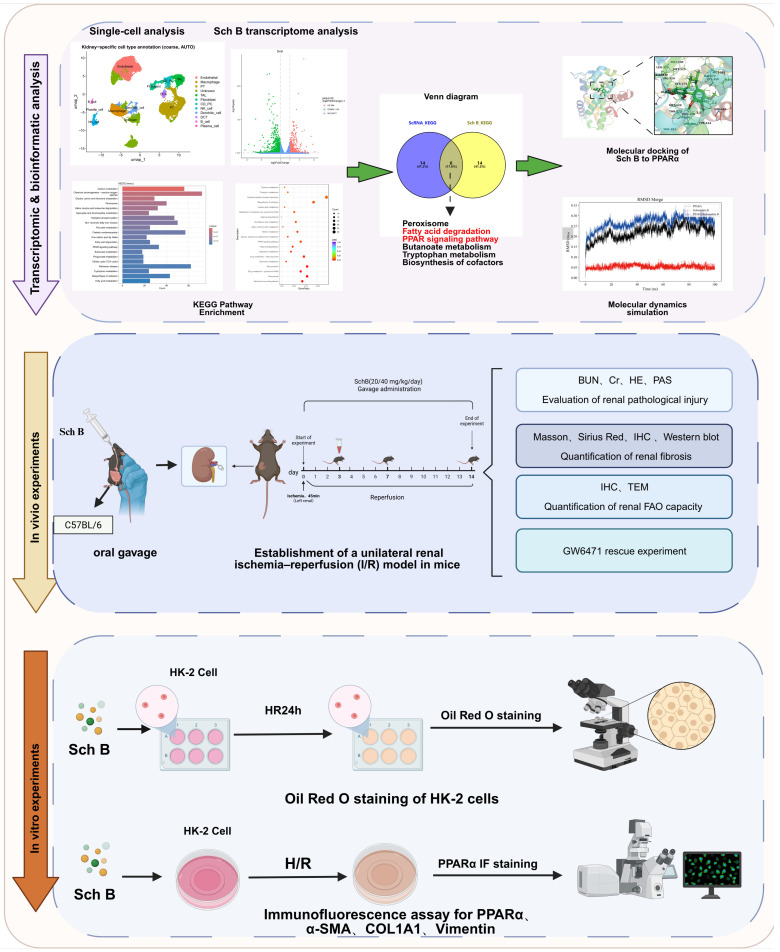
Overview of the experimental design and analytical workflow of the study. The study integrated single-cell RNA-seq and bulk transcriptomic analyses to identify metabolic pathways and PPARα-related signaling associated with renal ischemia–reperfusion injury (RIRI) and Schisandrin B (Sch B) intervention. In vivo, a unilateral renal I/R mouse model was established with Sch B pretreatment, followed by assessment of renal function, histopathological injury, fibrosis, fatty-acid oxidation (FAO)-related markers, and GW6471 rescue experiments. In vitro, HK-2 cells subjected to hypoxia/reoxygenation (H/R) were used for Oil Red O staining and immunofluores-cence analysis of PPARα, α-SMA, COL1A1, and vimentin. Molecular docking and mo-lecular dynamics simulations were performed to evaluate the interaction between Sch B and PPARα.

The overall experimental design and analytical workflow of the study are summarized in Figure 1.

## 3. Results

### 3.1. Single-Cell RNA-Seq Supports Proximal Tubular Injury-State Remodeling After Renal I/R

**Figure 2:**
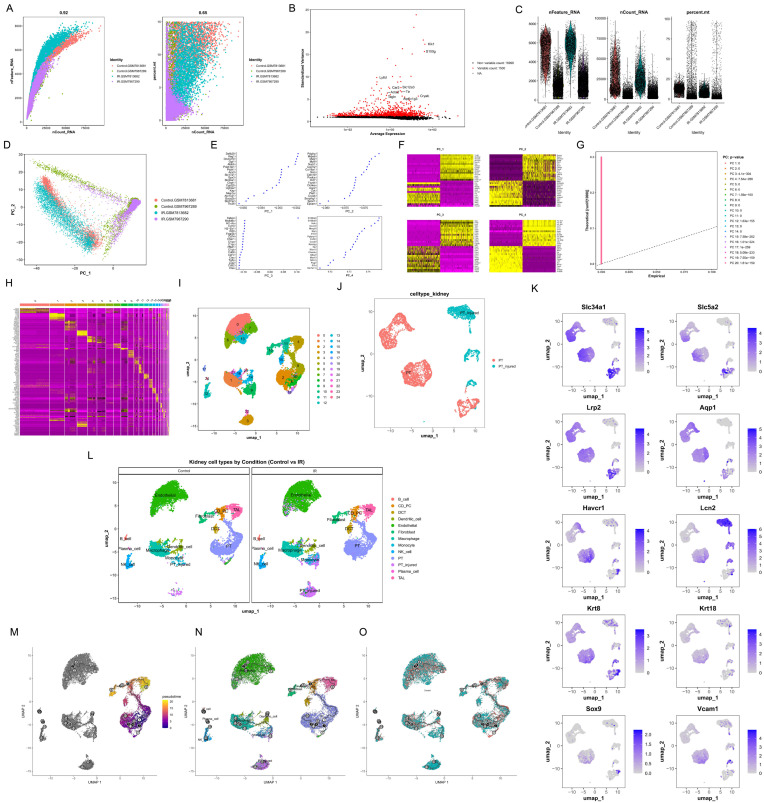
Single-cell RNA-seq analysis reveals proximal tubular injury and condition-dependent cellular remodeling after renal ischemia–reperfusion. (**A**) Scatter plots showing the relationships between nCount_RNA and nFeature_RNA, and between nCount_RNA and percent.mt across samples. (**B**) Identification of 1500 highly variable genes for downstream analysis. (**C**) Violin plots showing the distributions of nFeature_RNA, nCount_RNA, and percent.mt across samples after filtering. (**D**) PCA plot of integrated cells colored by sample identity. (**E**) Loading plots showing the top genes contributing to PC1–PC4. (**F**) Heatmaps showing expression patterns of genes with the strongest contributions to PC1–PC4. (**G**) JackStraw analysis indicating statistically significant principal components for downstream analysis. (**H**) Heatmap of cluster-enriched marker genes across identified cell clusters. (**I**) UMAP visualization of 25 transcriptionally distinct cell clusters. (**J**) UMAP highlighting proximal tubule (PT) and injured proximal tubule (PT_injured) populations. (**K**) Feature plots showing the expression of mature PT markers (Slc34a1, Slc5a2, Lrp2, Aqp1) and injury-associated markers (Havcr1, Lcn2, Krt8, Krt18, Sox9, Vcam1). (**L**) UMAP plots split by condition (Control vs. IR) showing cell-type distribution and compositional remodeling after injury. (**M**) Monocle3 pseudotime trajectory projected onto the UMAP. (**N**) Pseudotime trajectory colored by annotated cell type. (**O**) Pseudotime trajectory colored by condition, showing a shift of I/R cells toward later pseudotime states.

Single-cell RNA sequencing was performed to characterize cellular heterogeneity and injury-associated remodeling in control and ischemia–reperfusion (I/R) kidneys. After quality control, retained cells showed appropriate relationships among sequencing depth, detected gene number, and mitochondrial transcript proportion across samples (Figure 2A,C). A total of 1500 highly variable genes were selected for downstream analysis (Figure 2B). Principal component analysis (PCA), together with loading plots, heatmaps, and JackStraw statistics, supported the use of the leading principal components for subsequent clustering (Figure 2D–G).

Graph-based clustering resolved 25 transcriptionally defined clusters, which were annotated using canonical kidney cell markers and interpreted cautiously as cell types or cell states depending on marker distribution. (Figure 2H,I). To further define tubular injury states, we focused on proximal tubular populations and annotated mature proximal tubular and injury-associated proximal tubular states (Figure 2J). This annotation was supported by feature plots showing enrichment of mature PT markers, including Slc34a1, Slc5a2, Lrp2, and Aqp1, as well as injury-associated markers such as Havcr1, Lcn2, Krt8, Krt18, Sox9, and Vcam1 (Figure 2K).

When stratified by condition, the overall cellular architecture was preserved, but marked compositional changes were evident after I/R (Figure 2L). PT_injured cells were enriched in I/R kidneys, whereas differentiated PT cells were more prominent in controls. In parallel, immune and stromal populations, including macrophages, monocytes, dendritic cells, and fibroblasts, were expanded after I/R, indicating coordinated inflammatory and fibrogenic remodeling. Monocle3 trajectory analysis further revealed a continuous transition from PT cells toward PT_injured states along pseudotime (Figure 2M–O). Control cells were predominantly distributed at early pseudotime, whereas I/R cells shifted toward later pseudotime states. Together, these findings support the association of renal I/R with proximal tubular injury-state remodeling, dedifferentiation-like transcriptional changes, immune-cell expansion, and stromal activation.

### 3.2. Integrated Transcriptomic Analyses Support Metabolic Reprogramming and PPARα-Related Pathway Involvement After Renal I/R

**Figure 3:**
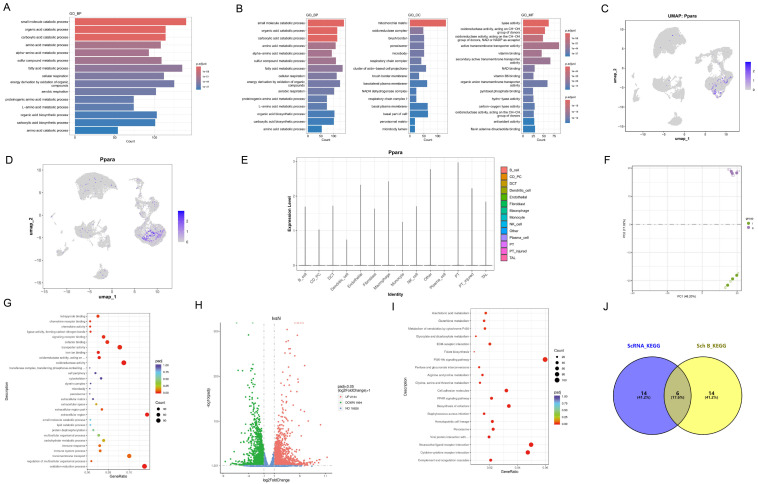
Integrated transcriptomic analyses support metabolic reprogramming and PPARα-related pathway involvement after renal I/R. (**A**) GO biological process enrichment analysis of the I/R-associated transcriptomic signature. (**B**) GO enrichment analysis across biological process (BP), cellular component (CC), and molecular function (MF) categories. (**C**) UMAP feature plot showing Ppara expression at the single-cell level. (**D**) Additional UMAP-based visualization of Ppara expression across kidney cell populations. (**E**) Cell type-resolved quantification of Ppara expression across annotated kidney populations. (**F**) Principal component analysis of the bulk transcriptomic dataset showing separation between comparison groups. (**G**) Bubble plot summarizing enriched GO terms related to metabolism, transport, and injury-associated biological responses. (**H**) Volcano plot showing differentially expressed genes in the bulk transcriptomic comparison. (**I**) KEGG pathway enrichment analysis of differentially expressed genes. (**J**) Venn diagram showing overlap between significantly enriched KEGG pathways derived from the single-cell I/R-associated signature (ScRNA_KEGG) and Sch B-responsive bulk transcriptomic pathways (Sch B_KEGG).

To investigate the molecular programs associated with renal ischemia–reperfusion injury (I/R) and their relationship to Sch B-responsive signaling, we integrated bulk and single-cell transcriptomic analyses. Gene Ontology (GO) enrichment analysis showed that the I/R-associated transcriptional signature was strongly enriched in small-molecule, organic-acid, amino-acid, and fatty-acid catabolic processes, indicating marked metabolic reprogramming after injury (Figure 3A). Expanded GO analyses further highlighted alterations in biological process, cellular component, and molecular function categories, including mitochondrial and peroxisomal compartments, brush-border structures, oxidoreductase activity, and transporter-related functions (Figure 3B,G).

At the single-cell level, Ppara displayed a heterogeneous expression pattern across renal cell populations (Figure 3C,D). UMAP-based visualization suggested that Ppara expression was mainly associated with tubular and selected stromal compartments rather than uniformly distributed across all cell types. Cell type-resolved quantification further showed relatively higher expression in PT-related populations, whereas immune subsets exhibited comparatively lower expression levels (Figure 3E). Given the overall modest expression intensity at the single-cell level, these findings were interpreted together with the bulk transcriptomic, histological, and functional data.

Bulk transcriptomic analysis showed clear separation between comparison groups on principal component analysis (Figure 3F), together with substantial differential gene expression visualized by volcano plotting (Figure 3H). KEGG enrichment analysis further identified pathways related to peroxisome function, fatty-acid metabolism, redox homeostasis, and PPAR signaling, indicating that Sch B-responsive transcriptional remodeling converges on lipid metabolic pathways relevant to renal I/R (Figure 3I). Consistently, comparison of significantly enriched KEGG pathways derived from the single-cell I/R-associated signature and Sch B-responsive bulk transcriptome revealed six shared pathways, including peroxisome, fatty acid degradation, PPAR signaling pathway, butanoate metabolism, tryptophan metabolism, and biosynthesis of cofactors (Figure 3J). Together, these transcriptomic findings provide supportive pathway-level evidence that renal I/R is accompanied by metabolic reprogramming and that Sch B-responsive pathways overlap with lipid metabolic and PPAR-related programs.

### 3.3. Schisandrin B Ameliorates Renal Dysfunction and Acute Tubular Injury After Renal Ischemia–Reperfusion

**Figure 4:**
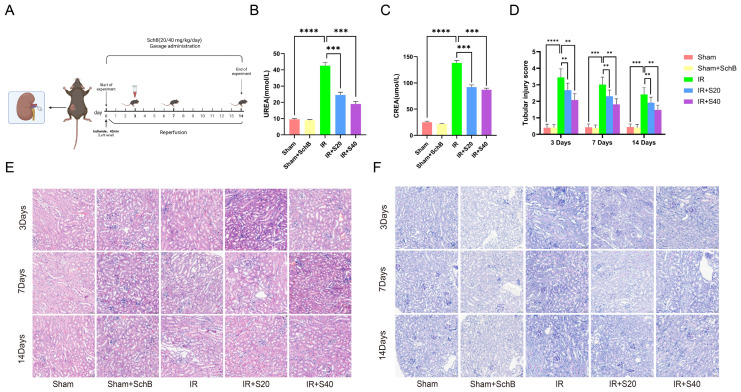
Schisandrin B ameliorates renal dysfunction and acute tubular injury after renal ischemia–reperfusion. (**A**) Schematic illustration of the in vivo experimental design. Mice were pretreated with Sch B (20 or 40 mg/kg/day) by oral gavage, subjected to unilateral renal ischemia for 45 min followed by reperfusion, and sacrificed at 3, 7, or 14 days after surgery. (**B**,**C**) Serum urea (**B**) and serum creatinine (**C**) levels in Sham, Sham+SchB, IR, IR+S20, and IR+S40 groups. (**D**) Semi-quantitative tubular injury scores at 3, 7, and 14 days after reperfusion. (**E**,**F**) Representative HE (**E**) and PAS (**F**) staining of kidney sections from the indicated groups and time points. Sch B treatment attenuated I/R-induced tubular injury and preserved renal histological architecture. Scale bar = 50 μm. Data are presented as mean ± SEM. ** *p* < 0.01, *** *p* < 0.001, **** *p* < 0.0001. *n* = 5 mice per group. Sham animals were regarded as the physiological baseline, and minor numerical differences between Sham and Sham+Sch B were not interpreted as biological improvement beyond baseline.

To evaluate the protective effect of Schisandrin B (Sch B) during the acute phase of renal ischemia–reperfusion injury (I/R), mice were pretreated with Sch B (20 or 40 mg/kg/day) and examined at 3, 7, and 14 days after reperfusion (Figure 4A). Renal function was markedly impaired after I/R, as reflected by increased serum urea and serum creatinine levels compared with the sham groups (Figure 4B,C). Sch B treatment significantly attenuated these changes, with the 40 mg/kg/day group showing a stronger renoprotective effect than the 20 mg/kg/day group.

Consistent with the biochemical data, tubular injury scores were substantially elevated in the I/R group at all examined time points, whereas Sch B treatment reduced the severity of tubular damage in a dose-dependent manner (Figure 4D). Histologically, hematoxylin–eosin (HE) and periodic acid–Schiff (PAS) staining showed marked tubular dilatation, epithelial degeneration, brush-border loss, and luminal cast formation after I/R. These pathological changes were alleviated in Sch B-treated kidneys, particularly in the IR+S40 group, indicating improved preservation of renal tubular architecture (Figure 4E,F). Together, these findings demonstrate that Sch B attenuates renal dysfunction and acute tubular injury after renal I/R.

### 3.4. Schisandrin B Attenuates Renal Fibrosis and Suppresses Fibrotic Marker Expression After Renal Ischemia–Reperfusion

**Figure 5:**
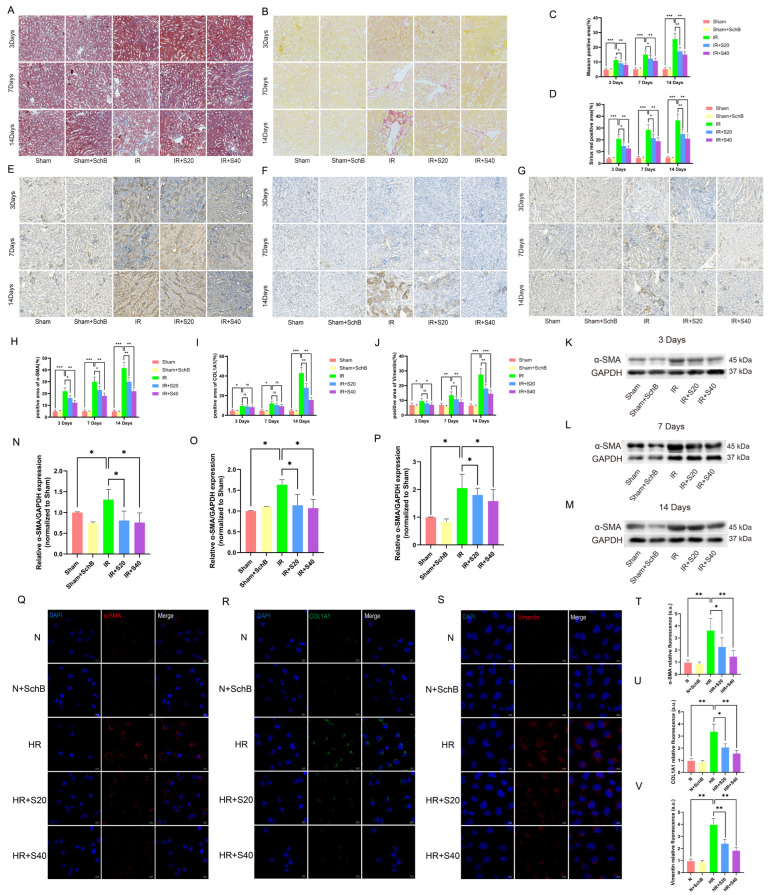
Schisandrin B attenuates renal fibrosis and suppresses fibrotic marker expression after renal ischemia–reperfusion. (**A**,**B**) Representative Masson trichrome (**A**) and Sirius Red (**B**) staining of kidney sections from Sham, Sham+SchB, IR, IR+S20, and IR+S40 groups at 3, 7, and 14 days after reperfusion. (**C**,**D**) Quantification of Masson-positive area (**C**) and Sirius Red-positive area (**D**). (**E**–**G**) Representative immunohistochemical staining for α-SMA (**E**), COL1A1 (**F**), and Vimentin (**G**) in kidney sections from the indicated groups and time points. (**H**–**J**) Quantification of α-SMA-positive area (**H**), COL1A1-positive area (**I**), and Vimentin-positive area (**J**). (**K**–**M**) Representative WWestern blot analysis of α-SMA and GAPDH at 3 days (**K**), 7 days (**L**), and 14 days (**M**) after reperfusion. (**N**–**P**) Densitometric quantification of relative α-SMA/GAPDH expression corresponding to 3 days (**N**), 7 days (**O**), and 14 days (**P**), normalized to the Sham group. (**Q**–**S**) Representative immunofluorescence staining of α-SMA (**Q**), COL1A1 (**R**), and Vimentin (**S**) in HK-2 cells under normoxic (N), normoxic + Sch B (N+SchB), hypoxia/reoxygenation (HR), HR+S20, and HR+S40 conditions. (**T**–**V**) Quantification of relative fluorescence intensity for α-SMA (**T**), COL1A1 (**U**), and Vimentin (**V**). Data are presented as mean ± SEM. Scale bar = 50 μm for histological and immunohistochemical staining; scale bar = 10 μm for immunofluorescence images. * *p* < 0.05, ** *p* < 0.01, *** *p* < 0.001. For Western blotting, *n* = 3 independent biological replicates per group, and each lane represents renal cortical lysate from one individual mouse. Uncropped western blot images and lane identities are provided in the Source Data file.

To determine whether Schisandrin B (Sch B) alleviates post-ischemic fibrotic remodeling, renal fibrosis was assessed by Masson trichrome staining, Sirius Red staining, immunohistochemistry, Western blotting, and in vitro immunofluorescence analysis. Masson and Sirius Red staining showed progressive interstitial collagen deposition after renal ischemia–reperfusion (I/R), whereas Sch B treatment markedly reduced fibrotic area at 3, 7, and 14 days after reperfusion (Figure 5A–D). This antifibrotic effect was more pronounced in the IR+S40 group than in the IR+S20 group.

Consistent with the histological findings, immunohistochemical staining demonstrated that the fibrotic markers α-SMA, COL1A1, and Vimentin were markedly increased in I/R kidneys and were substantially attenuated by Sch B treatment (Figure 5E–J). The reduction in fibrotic marker expression was most evident at later time points, indicating sustained suppression of fibrotic remodeling by Sch B.

Western blot analysis further confirmed that α-SMA protein expression was upregulated after I/R at 3, 7, and 14 days, whereas Sch B treatment significantly inhibited this increase (Figure 5K–P). In parallel, immunofluorescence analysis in HK-2 cells showed that hypoxia/reoxygenation (H/R) markedly increased α-SMA, COL1A1, and Vimentin fluorescence intensity, while Sch B treatment reduced the expression of these fibrotic markers in vitro (Figure 5Q–V). Collectively, these results indicate that Sch B attenuates renal fibrosis and reduces EMT-associated/profibrotic marker expression both in vivo and in vitro.

### 3.5. Schisandrin B Alleviates Lipid Accumulation, Preserves Mitochondrial Ultrastructure, and Restores FAO-Related Marker Expression After Renal I/R

**Figure 6:**
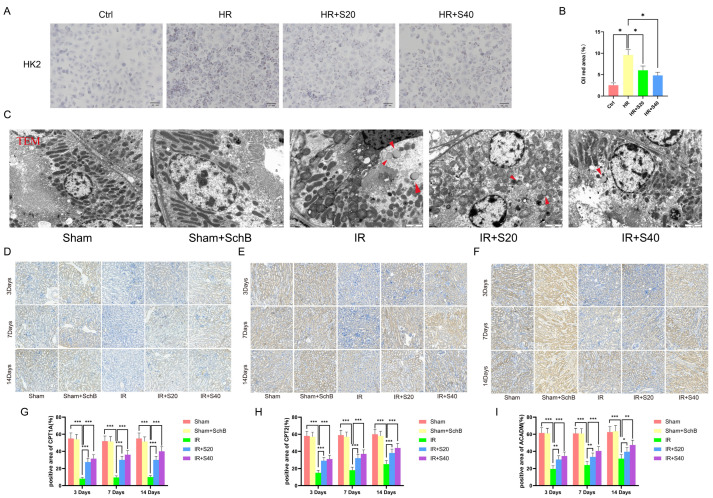
Schisandrin B alleviates lipid accumulation, preserves mitochondrial ultrastructure, and restores fatty acid oxidation after renal ischemia–reperfusion. (**A**) Representative Oil Red O staining of neutral lipid droplets in HK-2 cells under control conditions (Ctrl), hypoxia/reoxygenation (HR), and HR treated with 20 or 40 μM Sch B (HR+S20, HR+S40). (**B**) Quantification of Oil Red O–positive area in HK-2 cells. (**C**) Representative transmission electron microscopy images of renal tubular epithelial cells from Sham, Sham+SchB, IR, IR+S20, and IR+S40 groups, showing mitochondrial ultrastructure and lipid droplets (red arrows). (**D**–**F**) Representative immunohistochemical staining of CPT1A (**D**), CPT2 (**E**), and ACADM (**F**) in kidney sections from Sham, Sham+SchB, IR, IR+S20, and IR+S40 groups at 3, 7, and 14 days after reperfusion. (**G**–**I**) Semi-quantitative analysis of the positive staining area for CPT1A (**G**), CPT2 (**H**), and ACADM (**I**) at the indicated time points. Scale bar = 10 μm in (**A**), 2 μm in (**C**), and 50 μm in (**D**–**F**). Data are presented as mean ± SEM. * *p* < 0.05, ** *p* < 0.01, *** *p* < 0.001.

To determine whether the antifibrotic effect of Schisandrin B (Sch B) is associated with correction of lipid metabolic disturbances, we first examined neutral lipid accumulation in HK-2 cells subjected to hypoxia/reoxygenation (H/R). Oil Red O staining showed minimal lipid deposition in control cells, whereas H/R markedly increased cytoplasmic lipid accumulation (Figure 6A). Treatment with Sch B reduced lipid droplet accumulation, and quantitative analysis confirmed a significant decrease in Oil Red O–positive area in both the HR+S20 and HR+S40 groups compared with the H/R group (Figure 6B).

We next evaluated mitochondrial ultrastructure in vivo by transmission electron microscopy. Tubular epithelial cells from Sham and Sham+SchB kidneys exhibited relatively intact ultrastructure, whereas I/R kidneys showed obvious mitochondrial injury accompanied by increased electron-lucent lipid droplets (Figure 6C). Sch B pretreatment attenuated these ultrastructural abnormalities, with the IR+S40 group showing better preservation of mitochondrial integrity than the IR group.

To further assess whether Sch B restores renal fatty acid oxidation (FAO) after I/R, we examined the expression of key FAO-related proteins in kidney tissue. Immunohistochemistry showed that CPT1A, CPT2, and ACADM were strongly expressed in Sham kidneys but were markedly reduced after I/R at 3, 7, and 14 days (Figure 6D–F). Sch B pretreatment restored the staining intensity and positive area of all three proteins in a dose- and time-dependent manner, with the IR+S40 group generally showing stronger recovery than the IR+S20 group. Semi-quantitative analysis confirmed significant reductions in CPT1A-, CPT2-, and ACADM-positive areas in the I/R group, whereas Sch B significantly increased their expression compared with I/R alone (Figure 6G–I). These findings indicate that Sch B alleviates lipid accumulation and preserves mitochondrial ultrastructure in association with recovery of FAO-related marker expression after I/R injury.

### 3.6. Schisandrin B Restores PPARα Expression and Shows a Plausible Interaction with PPARα In Silico

**Figure 7:**
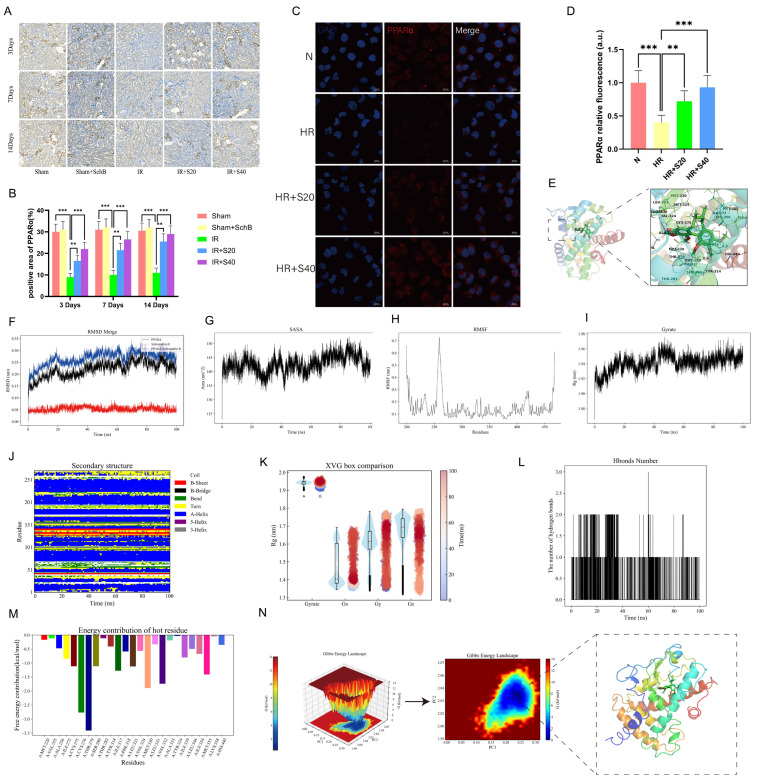
Schisandrin B restores PPARα expression and shows a plausible interaction with PPARα in silico. (**A**) Representative immunohistochemical staining of PPARα in kidney sections from Sham, Sham+SchB, IR, IR+S20, and IR+S40 groups at 3, 7, and 14 days after reperfusion. (**B**) Semi-quantitative analysis of the positive staining area for PPARα at the indicated time points. (**C**) Representative immunofluorescence staining of PPARα in HK-2 cells under normoxic (N), hypoxia/reoxygenation (HR), HR+S20, and HR+S40 conditions. (**D**) Quantification of relative PPARα fluorescence intensity. (**E**) Molecular docking model showing the predicted binding mode of Sch B within the PPARα ligand-binding pocket. (**F**) Root mean square deviation (RMSD) analysis of the simulated complex. (**G**) Solvent-accessible surface area (SASA) analysis during molecular dynamics simulation. (**H**) Root mean square fluctuation (RMSF) analysis of residue flexibility. (**I**) Radius of gyration (Rg) analysis during simulation. (**J**) Secondary-structure evolution of the PPARα complex during molecular dynamics simulation. (**K**) Comparative distribution of structural parameters derived from the simulation trajectory. (**L**) Time-dependent variation in hydrogen-bond number during simulation. (**M**) Free-energy contribution of hot residues involved in ligand binding. (**N**) Gibbs free energy landscape and representative low-energy conformation of the Sch B–PPARα complex. Scale bar = 50 μm in (**A**) and 10 μm in (**C**). Data are presented as mean ± SEM. ** *p* < 0.01, *** *p* < 0.001.

To further investigate whether the renoprotective effect of Schisandrin B (Sch B) is associated with PPARα signaling, we first examined PPARα expression in vivo and in vitro. Immunohistochemical staining showed that PPARα expression was markedly reduced in renal tissues after ischemia–reperfusion (I/R) at 3, 7, and 14 days, whereas Sch B pretreatment restored PPARα-positive staining in a dose-dependent manner (Figure 7A,B). Consistently, immunofluorescence analysis in HK-2 cells demonstrated that hypoxia/reoxygenation (H/R) markedly decreased PPARα fluorescence intensity, while Sch B treatment significantly increased PPARα expression compared with the H/R group (Figure 7C,D). These findings indicate that Sch B alleviates the I/R-associated suppression of PPARα.

To explore whether Sch B could plausibly interact with PPARα at the structural level, molecular docking was performed, followed by molecular dynamics simulation. Docking analysis suggested that Sch B could be accommodated within the ligand-binding pocket of PPARα and form multiple interactions with surrounding residues (Figure 7E). MD simulation further supported the stability of the Sch B–PPARα complex, as reflected by the overall behavior of the root mean square deviation (RMSD), solvent-accessible surface area (SASA), root mean square fluctuation (RMSF), and radius of gyration (Rg) during the simulation (Figure 7F–I). Secondary-structure analysis and structural parameter comparison further indicated that the complex remained conformationally stable throughout the simulation period (Figure 7J,K). In addition, hydrogen-bond occupancy, residue-level free-energy contribution, and Gibbs free energy landscape analyses supported persistent ligand–protein interaction and the presence of a relatively stable low-energy binding state (Figure 7L–N).Together, these results suggest that Sch B restores PPARα expression under I/R/H/R conditions and may form a plausible, structurally stable interaction with PPARα in silico. However, these computational findings should be interpreted as supportive structural evidence rather than proof of direct receptor activation.

### 3.7. GW6471 Partially Blunts the Antifibrotic and FAO Marker-Restorative Effects of Schisandrin B After Renal Ischemia–Reperfusion

**Figure 8:**
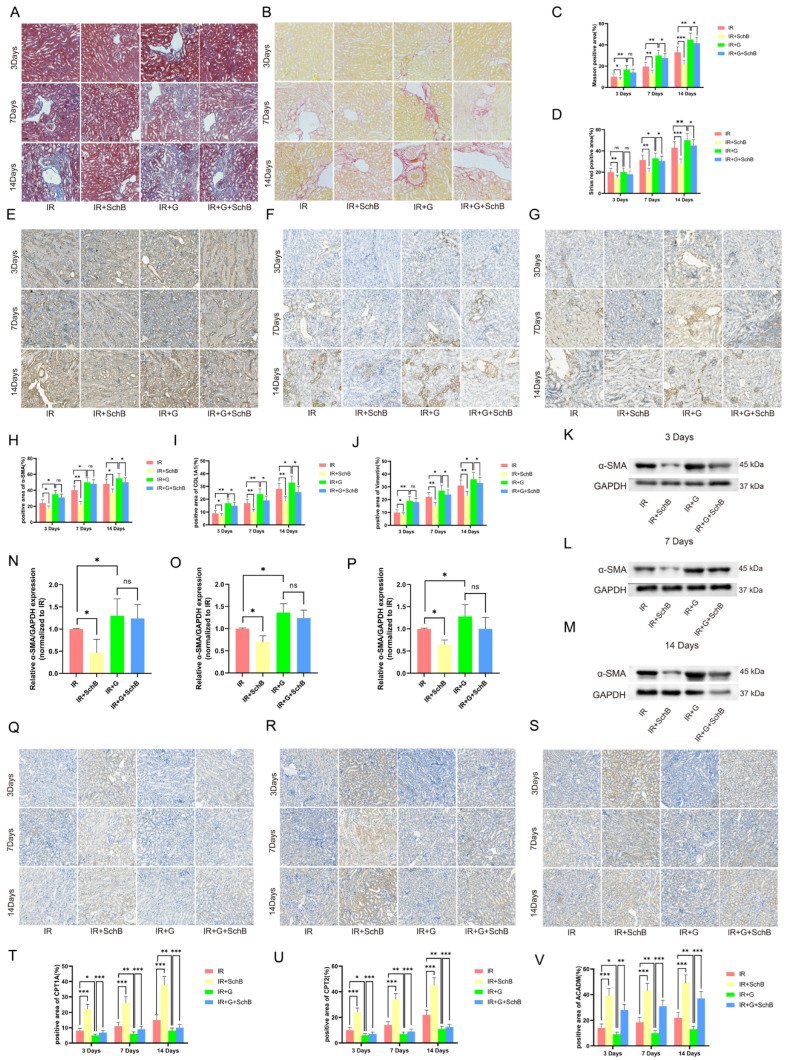
GW6471 partially blunts the antifibrotic and FAO marker-restorative effects of Schisandrin B after renal ischemia–reperfusion. (**A**,**B**) Representative Masson trichrome (**A**) and Sirius Red (**B**) staining of kidney sections from the IR, IR+SchB, IR+G, and IR+G+SchB groups at 3, 7, and 14 days after reperfusion. (**C**,**D**) Quantification of Masson-positive area (**C**) and Sirius Red-positive area (**D**). (**E**–**G**) Representative immunohistochemical staining for α-SMA (**E**), COL1A1 (**F**), and Vimentin (**G**) in kidney sections from the indicated groups and time points. (**H**–**J**) Quantification of α-SMA-positive area (**H**), COL1A1-positive area (**I**), and Vimentin-positive area (**J**). (**K**–**M**) Representative Western blot analysis of α-SMA and GAPDH at 3 days (**K**), 7 days (**L**), and 14 days (**M**) after reperfusion. (**N**–**P**) Densitometric quantification of relative α-SMA/GAPDH expression corresponding to 3 days (**N**), 7 days (**O**), and 14 days (**P**), normalized to the IR group. (**Q**–**S**) Representative immunohistochemical staining of CPT1A (**Q**), CPT2 (**R**), and ACADM (**S**) in kidney sections from the rescue experiment groups at 3, 7, and 14 days after reperfusion. (**T**–**V**) Semi-quantitative analysis of the positive staining area for CPT1A (**T**), CPT2 (**U**), and ACADM (**V**). Scale bar = 50 μm. Data are presented as mean ± SEM. ns, not significant; * *p* < 0.05, ** *p* < 0.01, *** *p* < 0.001. For Western blotting, *n* = 3 independent biological replicates per group, and each lane represents renal cortical lysate from one individual mouse. Uncropped western blot images and lane identities are provided in the Source Data file.

To further examine the functional involvement of PPARα signaling in the protective effect of Sch B, a GW6471 rescue experiment was performed in vivo. Histological assessment showed that Sch B reduced interstitial collagen deposition after renal ischemia–reperfusion (I/R), as demonstrated by decreased Masson trichrome- and Sirius Red-positive areas. In contrast, GW6471 treatment weakened this antifibrotic effect, and the IR+G and IR+G+SchB groups showed greater collagen accumulation than the IR+SchB group, particularly at 7 and 14 days after reperfusion (Figure 8A–D). These findings indicate that pharmacological inhibition of PPARα attenuates the fibrosis-limiting effect of Sch B.

Consistently, immunohistochemical staining showed that the fibrotic markers α-SMA, COL1A1, and Vimentin were reduced by Sch B treatment compared with the I/R group, whereas GW6471 blunted these reductions (Figure 8E–J). Although Sch B still showed a modest effect under some conditions, the overall suppressive effect on fibrotic marker expression was clearly weakened in the presence of GW6471.

Western blot analysis further confirmed that Sch B decreased α-SMA protein expression at 3, 7, and 14 days after I/R, whereas this inhibitory effect was attenuated when GW6471 was co-administered (Figure 8K–P). To evaluate whether the same dependency was observed in fatty acid oxidation (FAO)-related pathways, we also examined key FAO enzymes in rescue groups. Immunohistochemistry showed that CPT1A, CPT2, and ACADM expression was enhanced by Sch B treatment but was markedly reduced when GW6471 was introduced (Figure 8Q–V). Together, these findings indicate that PPARα inhibition blunts the antifibrotic and FAO-restorative effects of Sch B, supporting the idea that the renoprotective action of Sch B is at least partly mediated through PPARα-dependent metabolic regulation.

**Figure 9:**
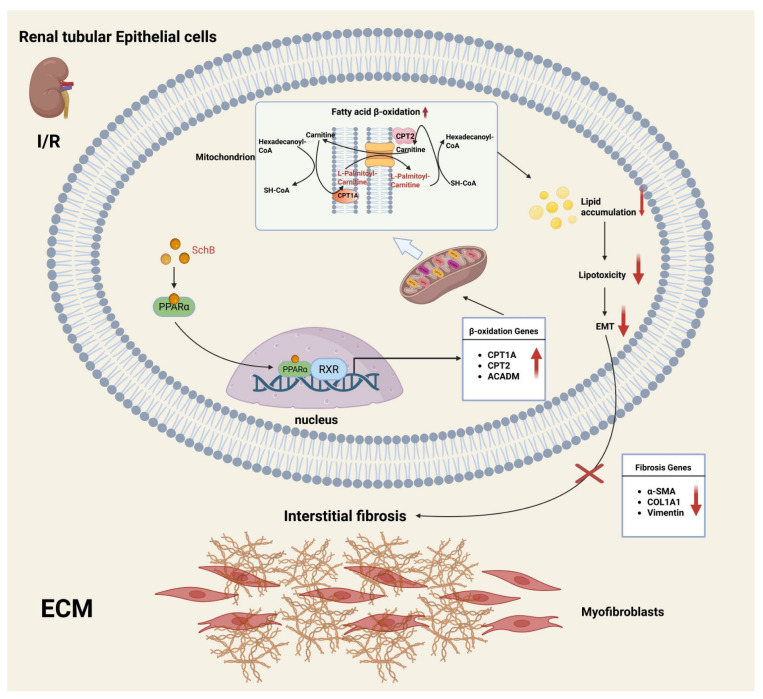
Proposed mechanism by which Schisandrin B attenuates renal fibrosis after renal ischemia–reperfusion injury. Schisandrin B (Sch B) is associated with restoration of a PPARα-related fatty acid β-oxidation (FAO) program in renal tubular epithelial cells, accompanied by increased expression of FAO-related genes, including CPT1A, CPT2, and ACADM. Enhanced FAO reduces lipid accumulation and lipotoxic stress, attenuates profibrotic marker expression (α-SMA, COL1A1, and Vimentin), and ultimately limits interstitial fibrosis after renal ischemia–reperfusion injury.

In Figure 9, the proposed model is showing that Sch B attenuates renal fibrotic remodeling after RIRI in association with restoration of PPARα-related tubular FAO signaling. Sch B may preserve FAO-related metabolic homeostasis, reduce intracellular lipid accumulation and lipotoxic stress, and thereby limit profibrotic remodeling. Direct receptor activation or ligand-level target engagement remains to be further validated.

## 4. Discussion

In the present study, we show that Schisandrin B (Sch B) markedly attenuates renal ischemia–reperfusion injury (RIRI) and subsequent fibrotic remodeling in association with reduced lipid accumulation, restoration of fatty acid oxidation (FAO)-related proteins, and recovery of PPARα signaling. In vivo, Sch B pretreatment improved renal function, as reflected by lower serum urea and creatinine levels, reduced tubular injury scores, and ameliorated histopathological damage. In parallel, Sch B decreased neutral lipid deposition, preserved mitochondrial ultrastructure, increased the expression of the FAO-related proteins CPT1A, CPT2, and ACADM, and suppressed the expression of profibrotic markers, including α-SMA, COL1A1, and vimentin. Importantly, pharmacologic inhibition with GW6471 blunted these protective effects, supporting the functional involvement of PPARα in the renoprotective and antifibrotic actions of Sch B. Taken together, our data support a model in which Sch B mitigates RIRI and limits progression toward fibrosis, at least in part, by restoring a PPARα-associated FAO program.

These findings extend previous work on Sch B in renal I/R. Earlier studies primarily emphasized its anti-inflammatory, antioxidant, and antiapoptotic activities. Xu et al. reported that Sch B alleviates RIRI through transcriptomic and pharmacological modulation of pathways involving AKT1, ALB, and CCL5, with downstream activation of PI3K/Akt signaling and attenuation of oxidative stress and tubular apoptosis [19]. More recently, Xu et al. further showed that Sch B regulates mitochondrial dynamics through AKT1 activation and mitochondrial targeting, thereby improving renal I/R injury [18]. In addition, Sch B has been linked to anti-inflammatory and stress-adaptive responses in other experimental settings, including AMPK/Nrf2-related cytoprotection and organ-protective effects beyond the kidney [13,14,16]. Sch B has also been reported to protect against myocardial ischemia/reperfusion injury through PI3K/Akt-associated mechanisms, further supporting its pleiotropic cytoprotective profile [17]. Rather than positioning these mechanisms as mutually exclusive, our data suggest that the renoprotective profile of Sch B likely reflects coordinated effects on mitochondrial integrity, stress adaptation, and energy metabolism. In this context, recovery of PPARα-related FAO appears to be a major downstream axis through which Sch B reduces lipotoxic stress and limits maladaptive repair.

The biological plausibility of this mechanism is strong. Renal proximal tubular epithelial cells are highly dependent on mitochondrial FAO for ATP production, and sustained suppression of FAO is increasingly recognized as a central feature of maladaptive repair after AKI [5,7,9]. When FAO is impaired, fatty acids are not efficiently utilized, leading to intracellular lipid accumulation, mitochondrial dysfunction, ATP depletion, and amplification of oxidative and inflammatory injury [7,9,11,12]. Thus, the reduction in Oil Red O-positive lipid droplets and the preservation of mitochondrial ultrastructure observed after Sch B treatment are not merely descriptive findings; rather, they are consistent with correction of a pathologic metabolic state closely linked to tubular vulnerability and fibrogenic remodeling. Our transcriptomic analyses further support this interpretation, because PPAR signaling, fatty acid metabolism, and peroxisome/mitochondria-related pathways emerged among the most responsive modules after Sch B treatment, while single-cell RNA-sequencing highlighted injury-associated metabolic reprogramming across tubular compartments. Together, these observations suggest that Sch B alleviates I/R-associated metabolic maladaptation and lipotoxic stress, which likely contributes to its downstream antifibrotic effect. It should be noted that the single-cell RNA-seq analysis was used to provide cell-type and cell-state context for renal I/R-associated remodeling. Because the analysis was based on public datasets and marker-based annotation, the PT and PT_injured labels should be interpreted as transcriptionally defined proximal tubular states rather than definitive independent cell types. Therefore, the single-cell results were considered supportive exploratory evidence and were not used as the sole basis for the mechanistic conclusion.

Among the pathways involved in renal lipid metabolism, PPARα is particularly relevant because it serves as a key transcriptional regulator of genes required for mitochondrial and peroxisomal FAO in the kidney [6,20]. Consistent with previous studies, we observed that renal I/R markedly suppressed PPARα expression together with its downstream FAO-related proteins, whereas Sch B treatment restored these changes. Earlier work by Sivarajah et al. showed that PPARα agonists such as clofibrate and WY-14643 reduce renal I/R injury [10], and other studies demonstrated that loss of PPARα signaling aggravates lipid deposition, tubular injury, and fibrosis, whereas preservation of the PPARα–FAO axis confers resistance to kidney injury [8,9,20,21]. In our study, the protective phenotype induced by Sch B was accompanied by increased renal PPARα expression and recovery of CPT1A, CPT2, and ACADM, supporting the interpretation that Sch B engages a PPARα-associated metabolic program. Notably, GW6471 weakened the ability of Sch B to suppress lipid accumulation, profibrotic marker expression, and fibrotic remodeling. This result provides pharmacologic support for the functional involvement of PPARα, although it does not by itself establish pathway exclusivity or prove that PPARα is the only mediator of the Sch B response. Our single-cell data are compatible with this interpretation, as Ppara expression was enriched mainly in tubular compartments and declined after I/R in association with expansion of injury-associated states, whereas Sch B partially restored this pattern. At the same time, the reductions in α-SMA, COL1A1, and vimentin are most conservatively interpreted as attenuation of EMT-associated/profibrotic remodeling rather than definitive proof of complete epithelial–mesenchymal transition blockade in a lineage-defined sense.

The rescue and in silico data should likewise be interpreted at an appropriate level of inference. In our hands, GW6471 consistently blunted, rather than uniformly and completely eliminated, the beneficial effects of Sch B on fibrosis-related readouts and FAO-related proteins, suggesting that PPARα is functionally important but not necessarily the exclusive determinant of protection. This is biologically reasonable given the pleiotropic nature of Sch B across multiple injury contexts [13,14,15,16,17,22]. In parallel, molecular docking and molecular dynamics simulations indicated that Sch B can adopt a plausible binding mode within the PPARα ligand-binding pocket and form a structurally stable predicted complex over the simulation period. These observations provide a useful structural rationale for possible target engagement, but they do not on their own establish direct binding in cells, receptor agonism, or target selectivity. Therefore, the current computational data should be regarded as supportive rather than definitive. Future studies using direct target-engagement or receptor-activation assays will be needed to determine whether Sch B acts as a bona fide PPARα ligand or instead modulates PPARα indirectly through upstream signaling, mitochondrial metabolic cues, or stress-response pathways.

Beyond the present study, the broader literature supports therapeutic targeting of the PPARα–FAO axis in AKI and kidney fibrosis. Pharmacologic or genetic interventions that preserve FAO generally improve renal outcomes, whereas inhibition of FAO aggravates injury and fibrotic progression [7,23,24,25,26]. Particularly relevant to our findings, oleoylethanolamide has been shown to attenuate acute-to-chronic kidney injury through proximal tubular PPARα-dependent mechanisms, and its protective effect is weakened in the setting of PPARα blockade or deficiency [27]. These parallels increase confidence that the phenotype we observed after Sch B treatment is biologically coherent and translationally relevant. The translational implications are potentially significant because renal I/R is central to ischemic AKI and highly relevant to kidney transplantation, where it contributes to delayed graft function and adverse long-term outcomes [4,28]. In addition, the protective effects of Sch B in hepatic ischemia–reperfusion models suggest that its cytoprotective actions may extend beyond the kidney, supporting broader organ-protective potential [15]. Because PPARα-targeting agents are already clinically familiar in other settings [26], the concept of time-limited metabolic intervention during or shortly after ischemic injury is attractive. Nonetheless, Sch B would require careful translational evaluation, especially in transplant recipients, because potential interactions with drug transporters and CYP3A-dependent immunosuppressive regimens may be clinically relevant [29,30]. More broadly, our findings intersect with the AKI-to-CKD transition, in which incomplete repair, persistent lipid metabolic derangement, and progressive fibrosis contribute to long-term functional decline [3,27].

From a translational perspective, the present findings may be relevant to clinical settings in which renal ischemia–reperfusion injury contributes to acute kidney injury, delayed graft function after kidney transplantation, and subsequent AKI-to-CKD progression. Because proximal tubular epithelial cells are highly dependent on mitochondrial fatty acid oxidation, metabolic preservation through PPARα-associated pathways may represent a rational strategy for limiting maladaptive repair after ischemic injury. However, the present study was designed primarily as a mechanistic preclinical investigation. Sch B was administered as a pretreatment, which may be more relevant to predictable ischemic conditions, such as kidney transplantation or planned renal surgery, than to unpredictable community-acquired AKI. Future studies should evaluate therapeutic administration after injury, dose–response optimization, pharmacokinetic behavior, long-term safety, and possible interactions with clinically used drugs, particularly immunosuppressive agents in the transplant setting.

Several limitations should be acknowledged. First, although GW6471 provided pharmacological evidence supporting the functional involvement of PPARα, the present study does not establish direct ligand-level activation or target engagement of PPARα by Sch B. Reporter assays, genetic loss-of-function models, and ligand-binding validation studies will be required to determine whether Sch B directly activates PPARα or indirectly modulates PPARα-associated metabolic signaling. Second, the animal sample size was limited, and no formal a priori power calculation was performed; therefore, the findings should be interpreted as mechanistic and exploratory rather than definitive. Third, although bulk RNA-seq and public single-cell RNA-seq analyses supported the involvement of lipid metabolic remodeling and tubular Ppara-related changes, these transcriptomic results should be regarded as supportive pathway-level evidence rather than independent functional validation. Fourth, changes in α-SMA, COL1A1, and vimentin were interpreted as evidence of EMT-associated/profibrotic remodeling, but they do not prove complete epithelial–mesenchymal transition in a lineage-defined manner. Finally, further studies are needed to evaluate pharmacokinetics, therapeutic dosing after established injury, long-term outcomes, and clinical applicability in kidney transplantation or AKI management.

## 5. Conclusions

In conclusion, the present study shows that Sch B alleviates renal ischemia–reperfusion injury and attenuates subsequent fibrotic remodeling in association with reduced lipid accumulation, preservation of mitochondrial ultrastructure, recovery of FAO-related proteins, and restoration of PPARα-associated signaling. Pharmacological inhibition with GW6471 supported the functional involvement of PPARα in this protective process. These findings suggest that restoration of tubular metabolic homeostasis is an important component of Sch B-associated renoprotection and provide a rationale for further evaluating Sch B and related metabolic interventions in ischemic kidney injury and AKI-to-CKD progression. Nevertheless, the precise mode of PPARα regulation by Sch B, including whether direct target engagement or receptor activation occurs, requires further investigation.

## Data Availability

The data that support the findings of this study are available from the corresponding author upon reasonable request.

## Supplementary Materials

The following supporting information can be downloaded at: https://www.mdpi.com/article/10.3390/biomedicines14061351/s1, Figures S1–S20: Original uncropped and cropped Western blot images for Figure 5; Figures S21–S43: Original uncropped and cropped Western blot images for Figure 8.

## Abbreviations

The following abbreviations are used in this manuscript:

ACADM	Medium-chain acyl-CoA dehydrogenase
AKI	Acute kidney injury
AKI-to-CKD	Acute kidney injury-to-chronic kidney disease
AKT1	AKT serine/threonine kinase 1
ALB	Albumin
alpha-SMA	Alpha-smooth muscle actin
ANOVA	Analysis of variance
ARRIVE	Animal Research: Reporting of In Vivo Experiments (guidelines)
ATP	Adenosine triphosphate
AVMA	American Veterinary Medical Association
BUN	Blood urea nitrogen
CCL5	C-C motif chemokine ligand 5
CKD	Chronic kidney disease
CPT1	Carnitine palmitoyltransferase 1
CPT1A	Carnitine palmitoyltransferase 1A
CPT2	Carnitine palmitoyltransferase 2
CYP3A	Cytochrome P450 3A
DAB	3,3′-Diaminobenzidine
DAPI	4′,6-diamidino-2-phenylindole
DCT	Distal convoluted tubule
DEGs	Differentially expressed genes
DGF	Delayed graft function
DMEM/F-12	Dulbecco’s modified Eagle medium/Nutrient Mixture F-12
DMSO	Dimethyl sulfoxide
EMT	Epithelial–mesenchymal transition
FAO	Fatty-acid beta-oxidation
FBS	Fetal bovine serum
GAFF	General AMBER Force Field
GAPDH	Glyceraldehyde-3-phosphate dehydrogenase
GO	Gene Ontology
GROMACS	GROningen MAchine for Chemical Simulations
GW6471	PPARalpha antagonist GW6471
H/R	Hypoxia/reoxygenation
HE	Hematoxylin and eosin
HISAT2	Hierarchical Indexing for Spliced Alignment of Transcripts 2
HK-2	Human proximal tubular epithelial cell line (HK-2)
HR	Hypoxia/reoxygenation
HRP	Horseradish peroxidase
I/R	Ischemia–reperfusion
IF	Immunofluorescence
IHC	Immunohistochemistry
JEOL	Japan Electron Optics Laboratory (JEOL)
KEGG	Kyoto Encyclopedia of Genes and Genomes
KIM-1	Kidney injury molecule-1
MD	Molecular dynamics
MM/GBSA	Molecular mechanics/generalized Born surface area
NGAL	Neutrophil gelatinase-associated lipocalin
NK	Natural killer (cells)
NP-40	Nonidet P-40
NPT	Constant particle number, pressure, and temperature ensemble
NVT	Constant particle number, volume, and temperature ensemble
OEA	Oleoylethanolamide
PAS	Periodic acid-Schiff
PBS	Phosphate-buffered saline
PCA	Principal component analysis
PPAR	Peroxisome proliferator-activated receptor
PPARalpha	Peroxisome proliferator-activated receptor-alpha
PT	Proximal tubule
PVDF	Polyvinylidene fluoride
PyMOL	PyMOL Molecular Graphics System
RCSB	Research Collaboratory for Structural Bioinformatics
RESP	Restrained electrostatic potential
Rg	Radius of gyration
RIN	RNA integrity number
RIRI	Renal ischemia–reperfusion injury
RMSD	Root-mean-square deviation
RMSF	Root-mean-square fluctuation
RNA	Ribonucleic acid
RNA-seq	RNA sequencing
SASA	Solvent-accessible surface area
Scr	Serum creatinine
scRNA-seq	Single-cell RNA sequencing
SD	Standard deviation
SDS-PAGE	Sodium dodecyl sulfate-polyacrylamide gel electrophoresis
TAL	Thick ascending limb
TBS-T	Tris-buffered saline with Tween 20
TEM	Transmission electron microscopy
TIP3P	Transferable Intermolecular Potential 3-Point (water model)
TRIzol	TRIzol reagent
UMAP	Uniform Manifold Approximation and Projection
UMI	Unique molecular identifier

## References

[1] Li C., Yu Y., Zhu S., Hu Y., Ling X., Xu L., Zhang H., Guo K. (2024). The emerging role of regulated cell death in ischemia and reperfusion-induced acute kidney injury: Current evidence and future perspectives. Cell Death Discov..

[2] Granata S., Votrico V., Spadaccino F., Catalano V., Netti G.S., Ranieri E., Stallone G., Zaza G. (2022). Oxidative Stress and Ischemia/Reperfusion Injury in Kidney Transplantation: Focus on Ferroptosis, Mitophagy and New Antioxidants. Antioxidants.

[3] Koh E.S., Chung S. (2024). Recent Update on Acute Kidney Injury-to-Chronic Kidney Disease Transition. Yonsei Med. J..

[4] Li M.T., Ramakrishnan A., Yu M., Daniel E., Sandra V., Sanichar N., King K., Stevens J., Husain S.A., Mohan S. (2023). Effects of Delayed Graft Function on Transplant Outcomes: A Meta-analysis. Transplant. Direct.

[5] Gewin L.S. (2021). Sugar or Fat? Renal Tubular Metabolism Reviewed in Health and Disease. Nutrients.

[6] Gao J., Gu Z. (2022). The Role of Peroxisome Proliferator-Activated Receptors in Kidney Diseases. Front. Pharmacol..

[7] Kang H.M., Ahn S.H., Choi P., Ko Y.-A., Han S.H., Chinga F., Park A.S.D., Tao J., Sharma K., Pullman J. (2015). Defective fatty acid oxidation in renal tubular epithelial cells has a key role in kidney fibrosis development. Nat. Med..

[8] Li J., Yang Y., Li Q., Wei S., Zhou S., Yu W., Xue L., Zhou L., Shen L., Lu G. (2022). STAT6 contributes to renal fibrosis by modulating PPARα-mediated tubular fatty acid oxidation. Cell Death Dis..

[9] Chung K.W., Lee E.K., Lee M.K., Oh G.T., Yu B.P., Chung H.Y. (2018). Impairment of PPARα and the Fatty Acid Oxidation Pathway Aggravates Renal Fibrosis during Aging. J. Am. Soc. Nephrol..

[10] Sivarajah A., Chatterjee P.K., Hattori Y., Brown P.A., Stewart K.N., Mota-Filipe H., Cuzzocrea S., Thiemermann C. (2002). Agonists of peroxisome-proliferator activated receptor-alpha (clofibrate and WY14643) reduce renal ischemia/reperfusion injury in the rat. Med. Sci. Monit..

[11] Jao T.M., Nangaku M., Wu C.H., Sugahara M., Saito H., Maekawa H., Ishimoto Y., Aoe M., Inoue T., Tanaka T. (2019). ATF6α downregulation of PPARα promotes lipotoxicity-induced tubulointerstitial fibrosis. Kidney Int..

[12] Otunla A.A., Shanmugarajah K., Davies A.H., Shalhoub J. (2024). Lipotoxicity and immunometabolism in ischemic acute kidney injury: Current perspectives and future directions. Front. Pharmacol..

[13] Luo W., Lin K., Hua J., Han J., Zhang Q., Chen L., Khan Z.A., Wu G., Wang Y., Liang G. (2022). Schisandrin B Attenuates Diabetic Cardiomyopathy by Targeting MyD88 and Inhibiting MyD88-Dependent Inflammation. Adv. Sci..

[14] Zhao B., Li G.P., Peng J.J., Ren L.H., Lei L.C., Ye H.M., Wang Z.Y., Zhao S. (2021). Schizandrin B attenuates hypoxia/reoxygenation injury in H9c2 cells by activating the AMPK/Nrf2 signaling pathway. Exp. Ther. Med..

[15] Li X., Zhao Y., Gong S., Song T., Ge J., Li J., Zhang J., Fu K., Zheng Y., Ma L. (2023). Schisandrin B ameliorates acute liver injury by regulating EGFR-mediated activation of autophagy. Bioorg. Chem..

[16] Zhu W., Luo W., Han J., Zhang Q., Ji L., Samorodov A.V., Pavlov V.N., Zhuang Z., Yang D., Yin L. (2023). Schisandrin B protects against LPS-induced inflammatory lung injury by targeting MyD88. Phytomedicine.

[17] Zhao X., Xiang Y., Cai C., Zhou A., Zhu N., Zeng C. (2018). Schisandrin B protects against myocardial ischemia/reperfusion injury via the PI3K/Akt pathway in rats. Mol. Med. Rep..

[18] Xu C., Wang H., Wang H., Man J., Deng Y., Li Y., Cheng K., Niu J., Gui H., Fu S. (2025). Schisandrin B regulates mitochondrial dynamics via AKT1 activation and mitochondrial targeting to ameliorate renal ischemia-reperfusion injury. Phytomedicine.

[19] Xu C., Deng Y., Man J., Wang H., Che T., Ding L., Yang L. (2024). Unveiling the Renoprotective Mechanisms of Schisandrin B in Ischemia-Reperfusion Injury Through Transcriptomic and Pharmacological Analysis. Drug Des. Dev. Ther..

[20] Cheng C.F., Chen H.H., Lin H. (2010). Role of PPARα and Its Agonist in Renal Diseases. PPAR Res..

[21] Chen L., Sha M.L., Chen F.T., Jiang C.Y., Li D., Xu C.L., Pan D.S., Xu Z.J., Yang Q.L., Xia S.J. (2023). Upregulation of KLF14 expression attenuates kidney fibrosis by inducing PPARα-mediated fatty acid oxidation. Free Radic. Biol. Med..

[22] Zhang W., Sun Z., Meng F. (2017). Schisandrin B Ameliorates Myocardial Ischemia/Reperfusion Injury Through Attenuation of Endoplasmic Reticulum Stress-Induced Apoptosis. Inflammation.

[23] Xu S., Jia P., Fang Y., Jin J., Sun Z., Zhou W., Li J., Zhang Y., Wang X., Ren T. (2022). Nuclear farnesoid X receptor attenuates acute kidney injury through fatty acid oxidation. Kidney Int..

[24] Li S., Wu P., Yarlagadda P., Vadjunec N.M., Praia A.D., Harris R.A., Portilla D. (2004). PPAR alpha ligand protects during cisplatin-induced acute renal failure by preventing inhibition of renal FAO and PDC activity. Am. J. Physiol. Ren. Physiol..

[25] Iwaki T., Bennion B.G., Stenson E.K., Lynn J.C., Otinga C., Djukovic D., Fartery D., Fei L., Wong H.R., Liles C. (2019). PPARα contributes to protection against metabolic and inflammatory derangements associated with acute kidney injury in experimental sepsis. Physiol. Rep..

[26] Davis T.M.E., Ting R., Best J.D., Donoghoe M.W., Drury P.L., Sullivan D.R., Jenkins A.J., O’Connell R.L., Whiting M.J., Glasziou P.P. (2011). Effects of fenofibrate on renal function in patients with type 2 diabetes mellitus: The Fenofibrate Intervention and Event Lowering in Diabetes (FIELD) Study. Diabetologia.

[27] Comella F., Lama A., Pirozzi C., Annunziata C., Piegari G., Sodano F., Melini S., Paciello O., Lago Paz F., Meli R. (2024). Oleoylethanolamide attenuates acute-to-chronic kidney injury: In vivo and in vitro evidence of PPAR-α involvement. Biomed. Pharmacother..

[28] Siedlecki A., Irish W., Brennan D.C. (2011). Delayed graft function in the kidney transplant. Am. J. Transplant..

[29] Qin X.L., Chen X., Zhong G.P., Fan X.M., Wang Y., Xue X.P., Wang Y., Huang M., Bi H.C. (2014). Effect of Tacrolimus on the pharmacokinetics of bioactive lignans of Wuzhi tablet (*Schisandra sphenanthera* extract) and the potential roles of CYP3A and P-gp. Phytomedicine.

[30] Li W.L., Xin H.W., Yu A.R., Wu X.C. (2013). In vivo effect of Schisandrin B on cytochrome P450 enzyme activity. Phytomedicine.

