# Supplementary material for: Schisandrin B Attenuates Renal Fibrotic Remodeling in Association with Restoration of a PPARα-Related Tubular Fatty-Acid Oxidation Program
Source: Biomedicines. 2026 Jun 15;14(6):1351. doi: 10.3390/biomedicines14061351 (PMC13297011; doi:10.3390/biomedicines14061351)
Supplement: Supplementary file 1 [file biomedicines-14-01351-s001.zip › biomedicines-4301470-supplementary.pdf]

Supplementary Figures S1-S20

Figure S1–S20: Original uncropped and cropped Western blot images for Figure 5

3 days

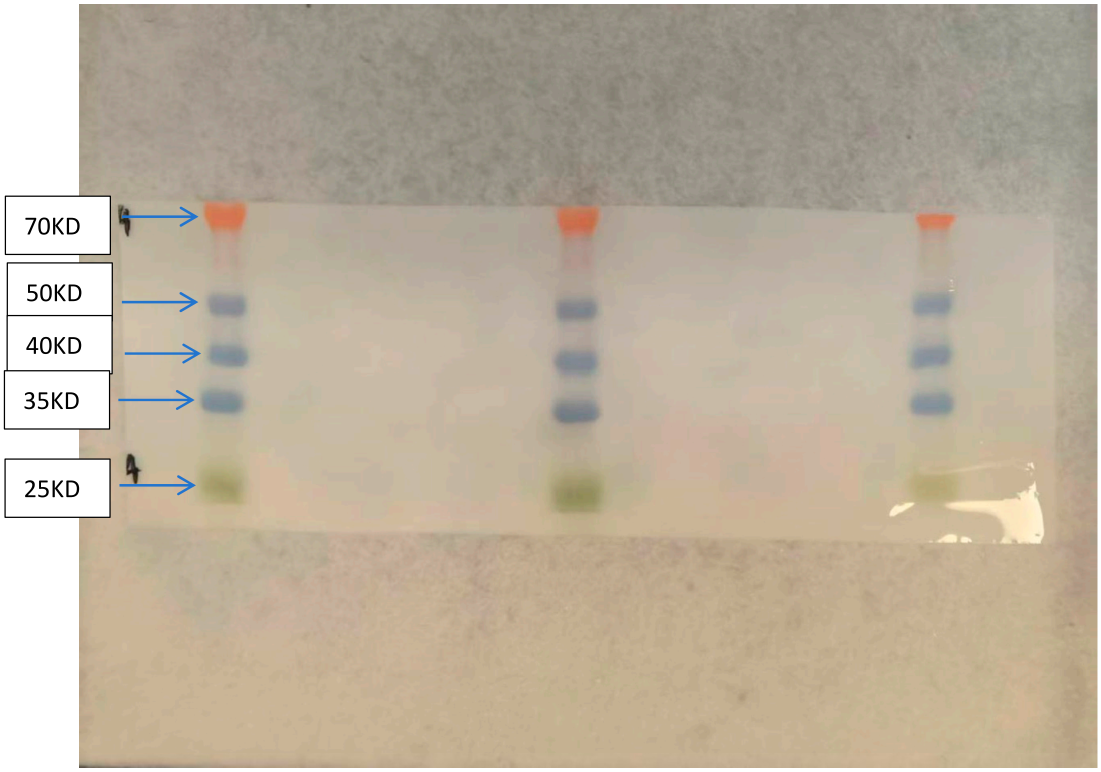

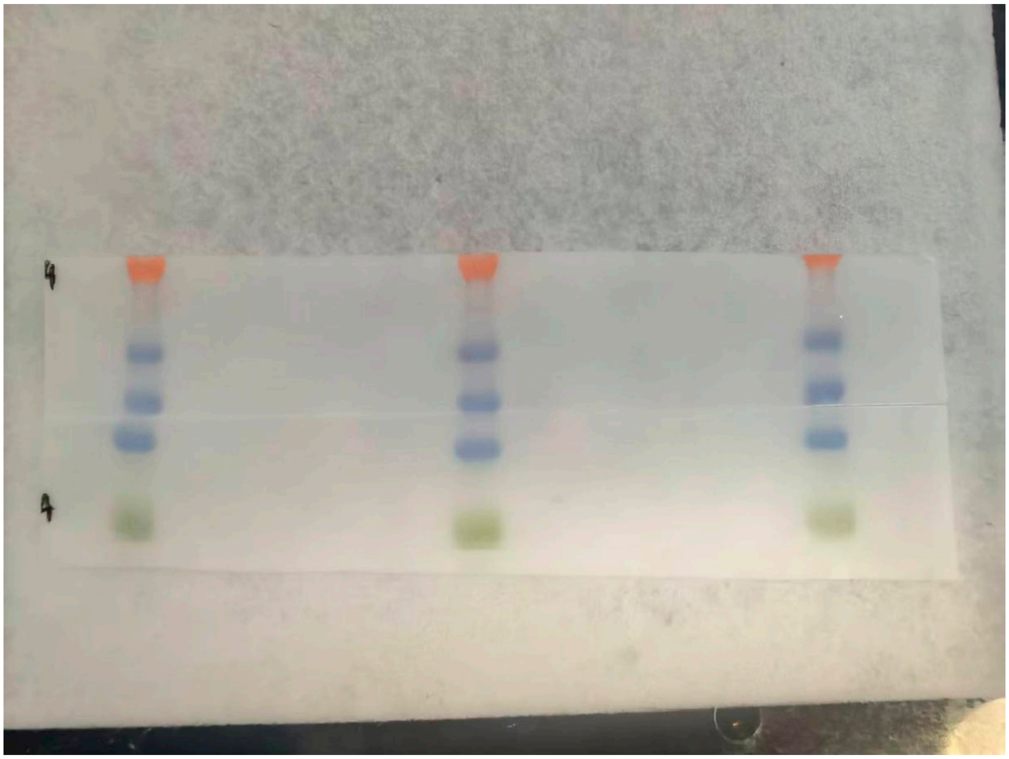

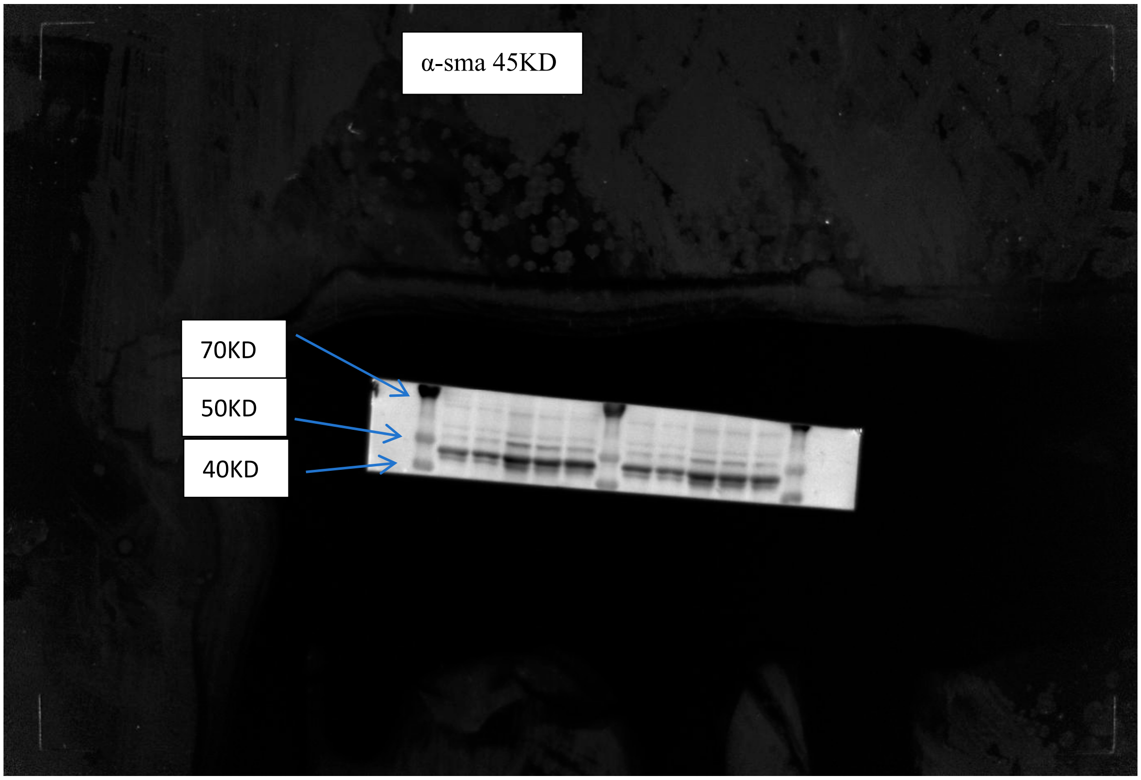
α-sma 45KD
70KD
50KD
40KD

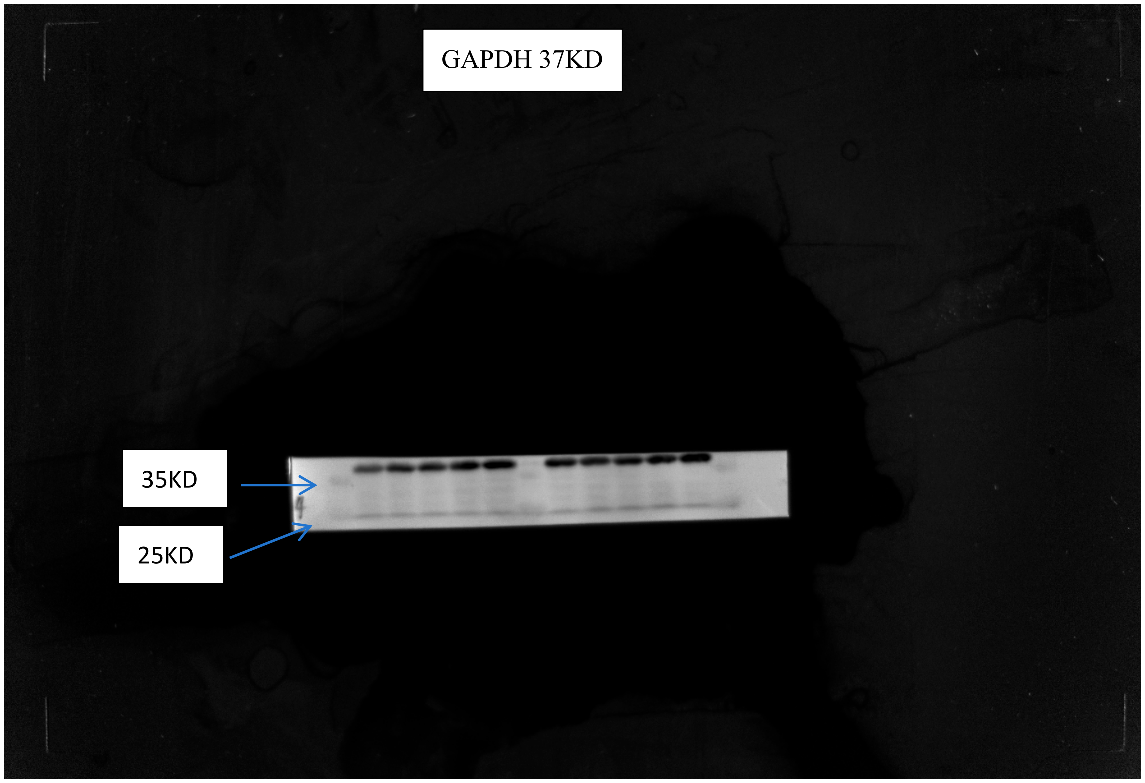

3 days-7 days

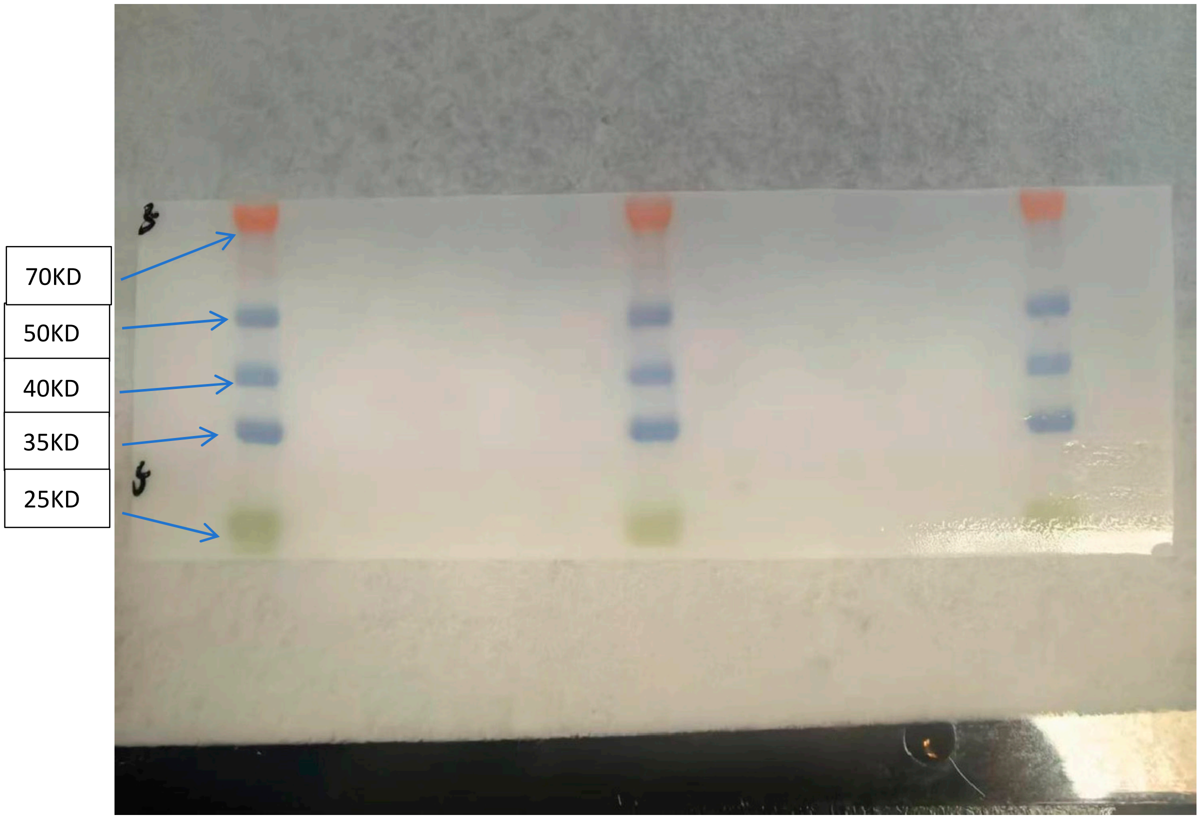
70KD
50KD
40KD
35KD
25KD

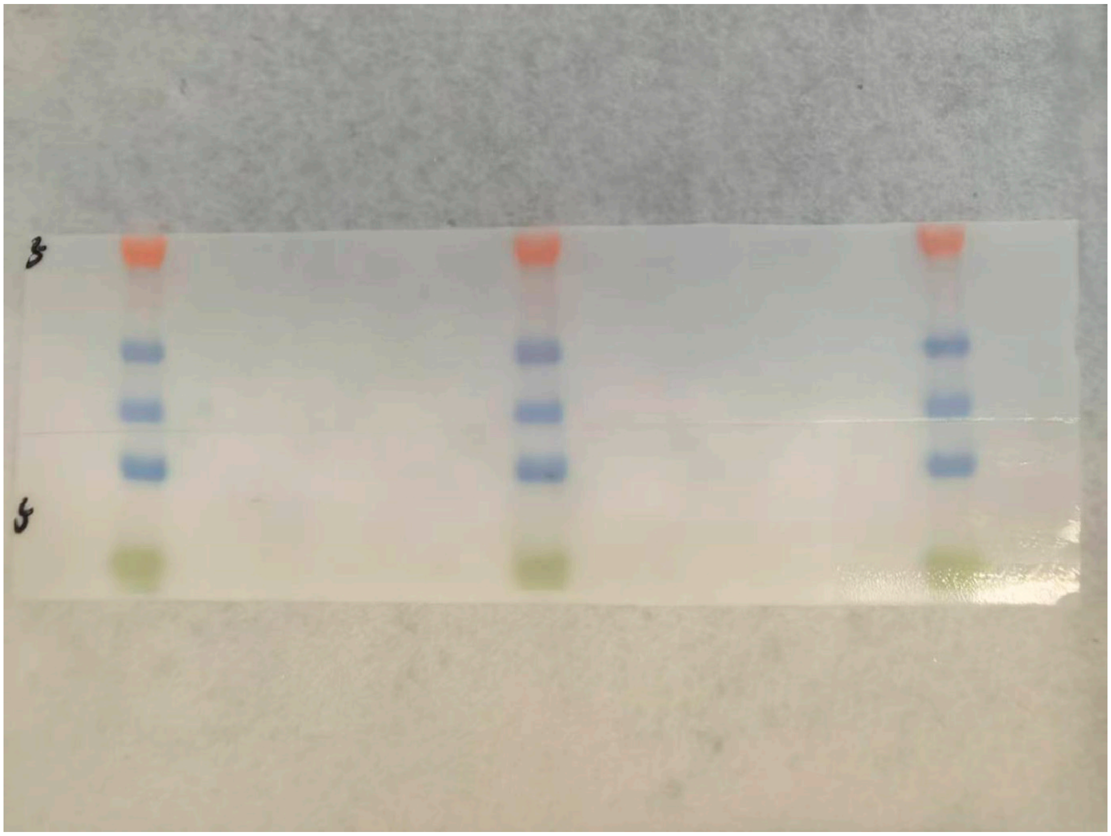

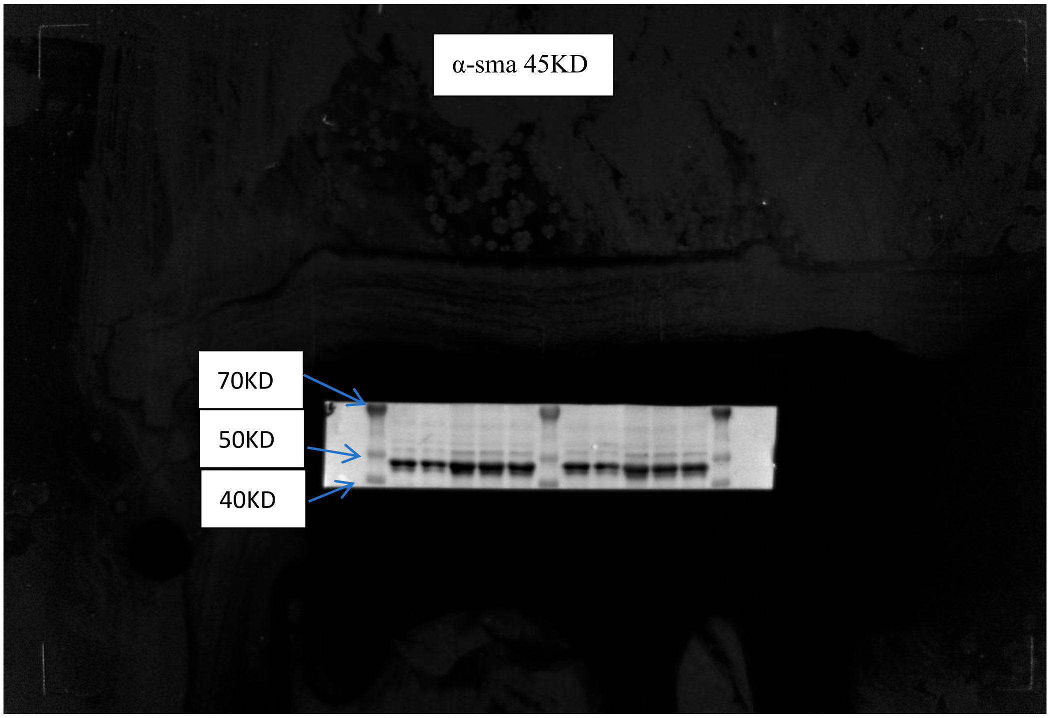
α-sma 45KD
70KD
50KD
40KD

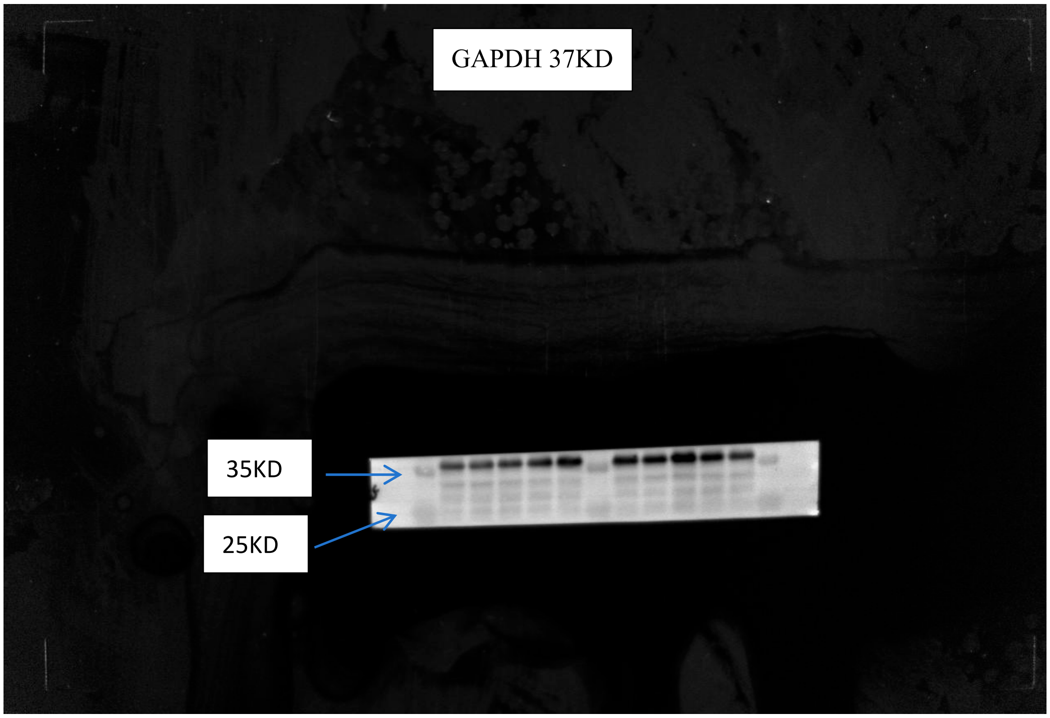
GAPDH 37KD
35KD
25KD

7 days

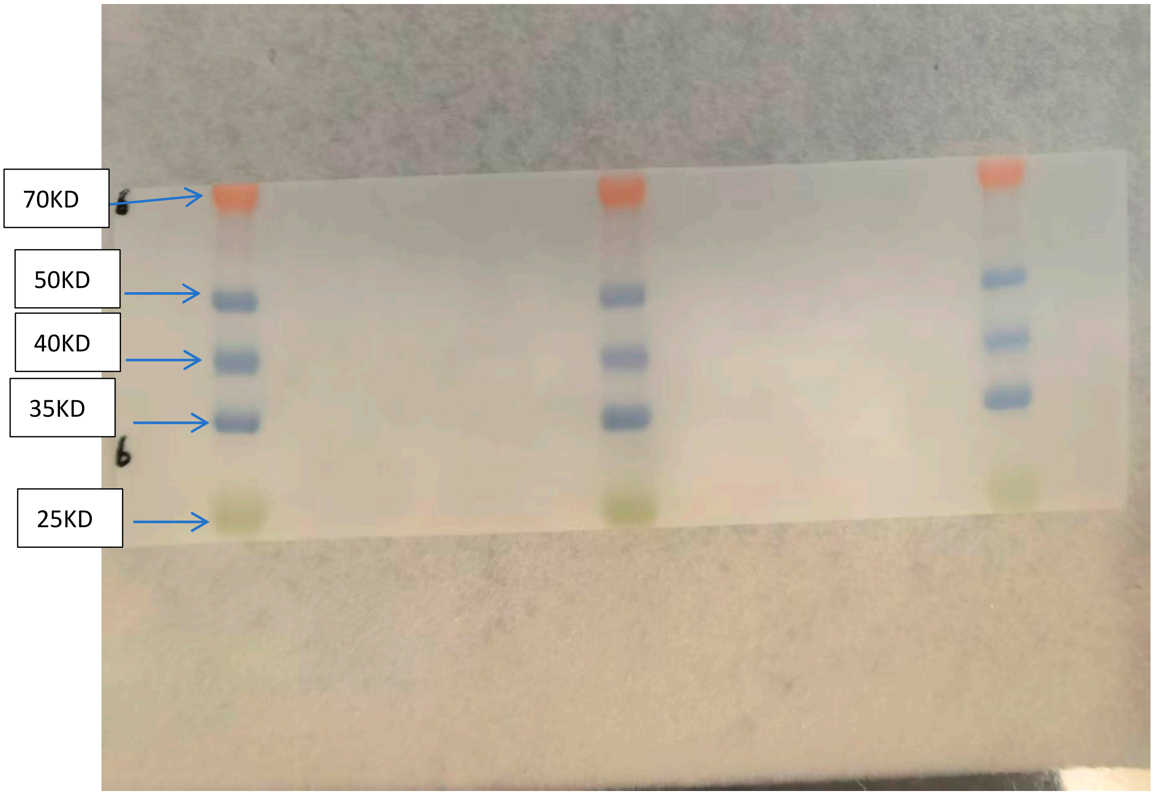

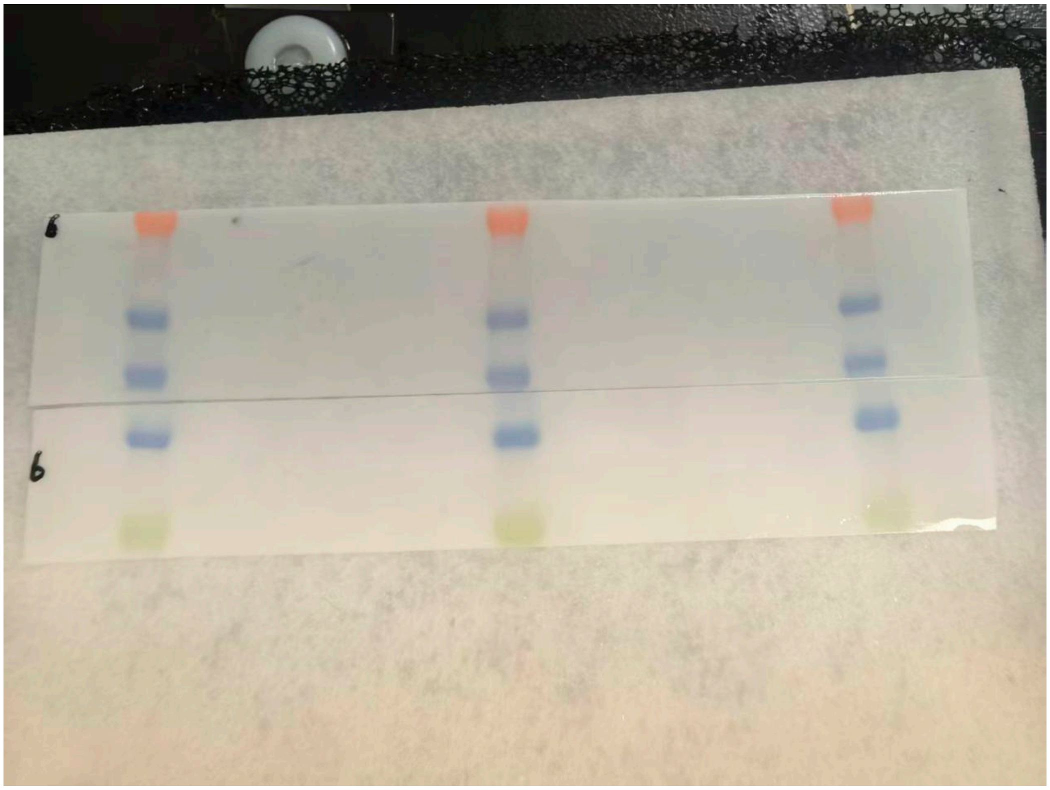

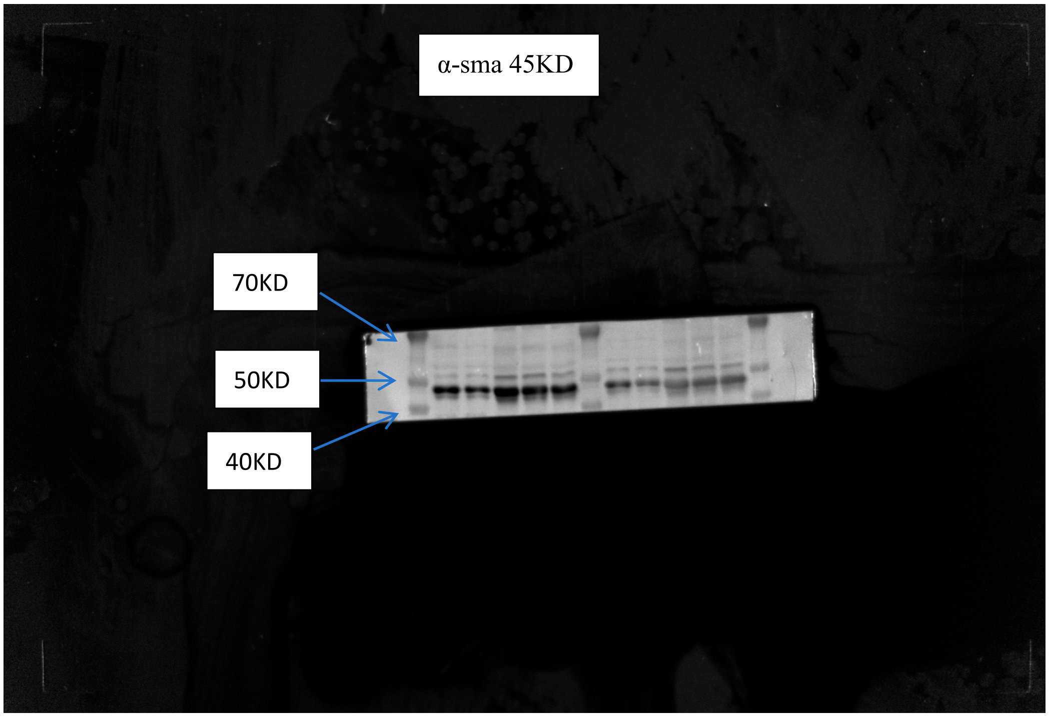
α-sma 45KD
70KD
50KD
40KD

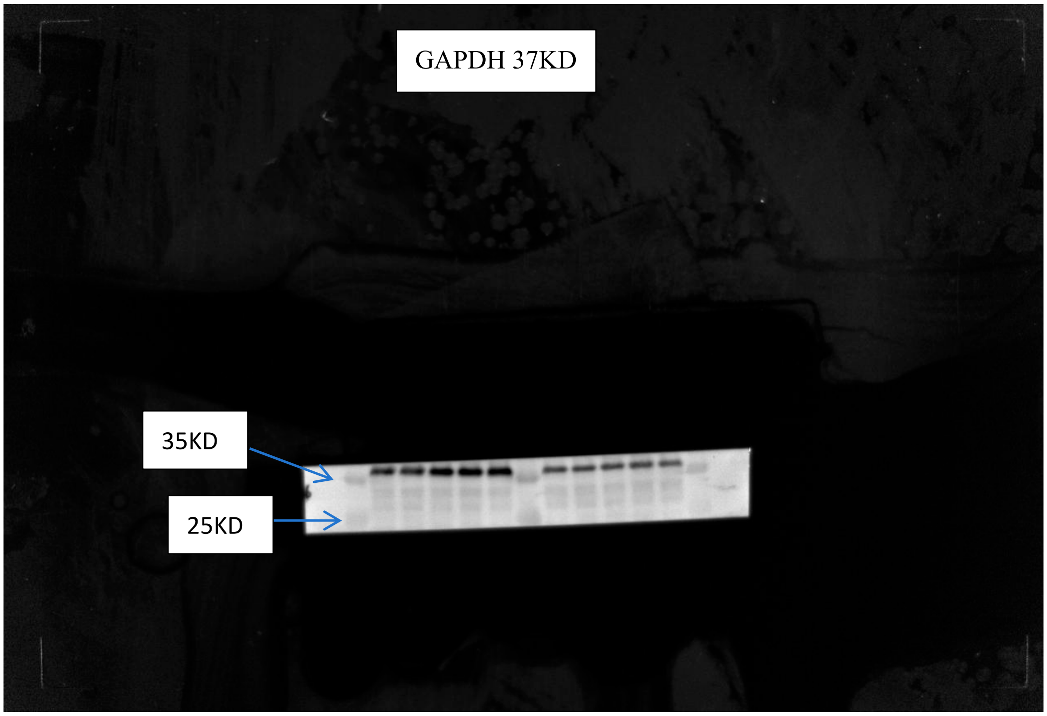
GAPDH 37KD
35KD
25KD

14 days

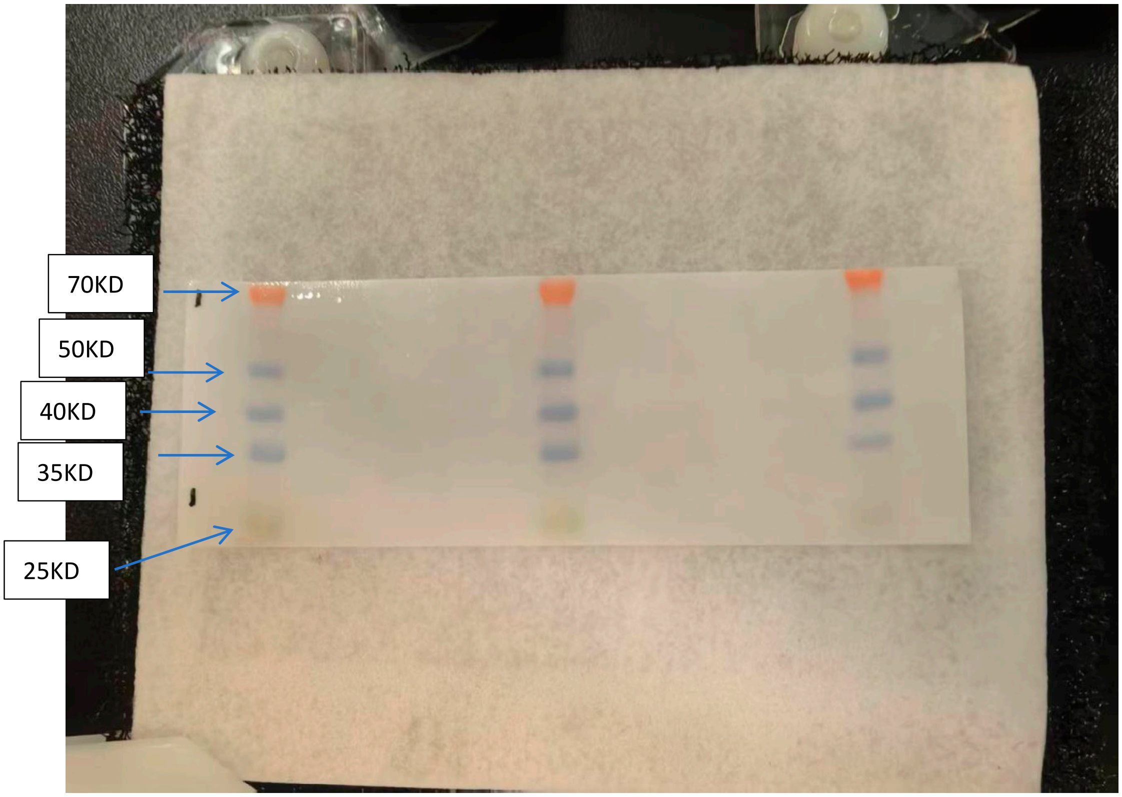

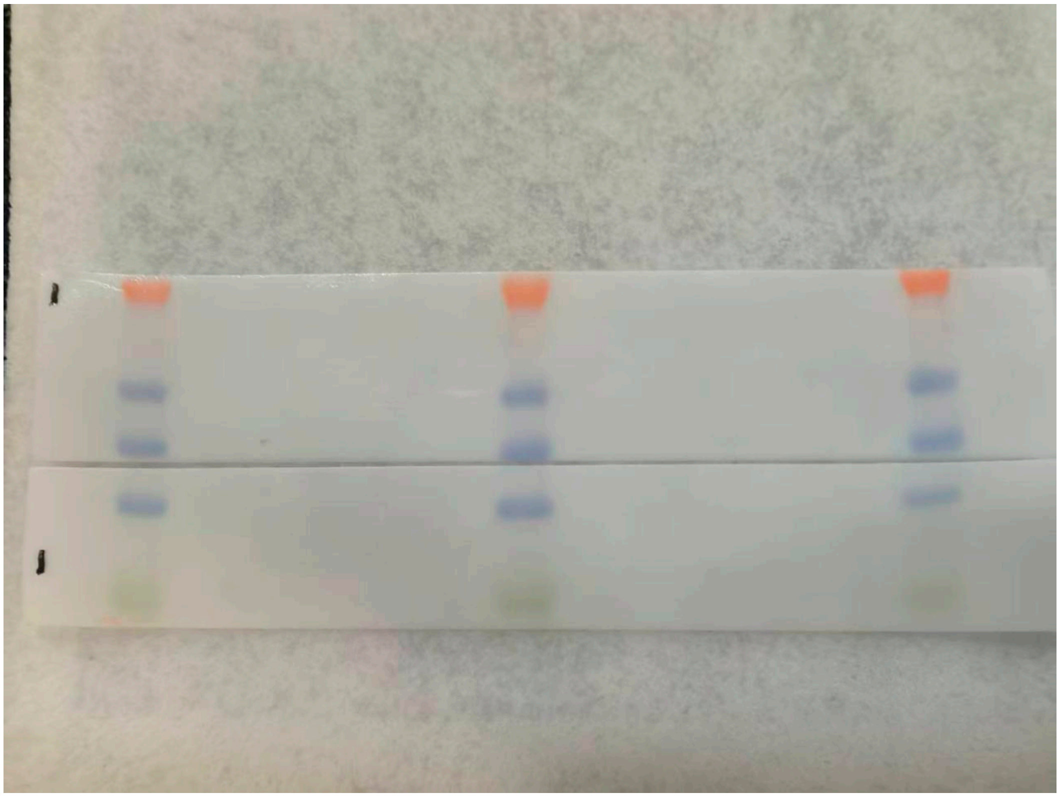

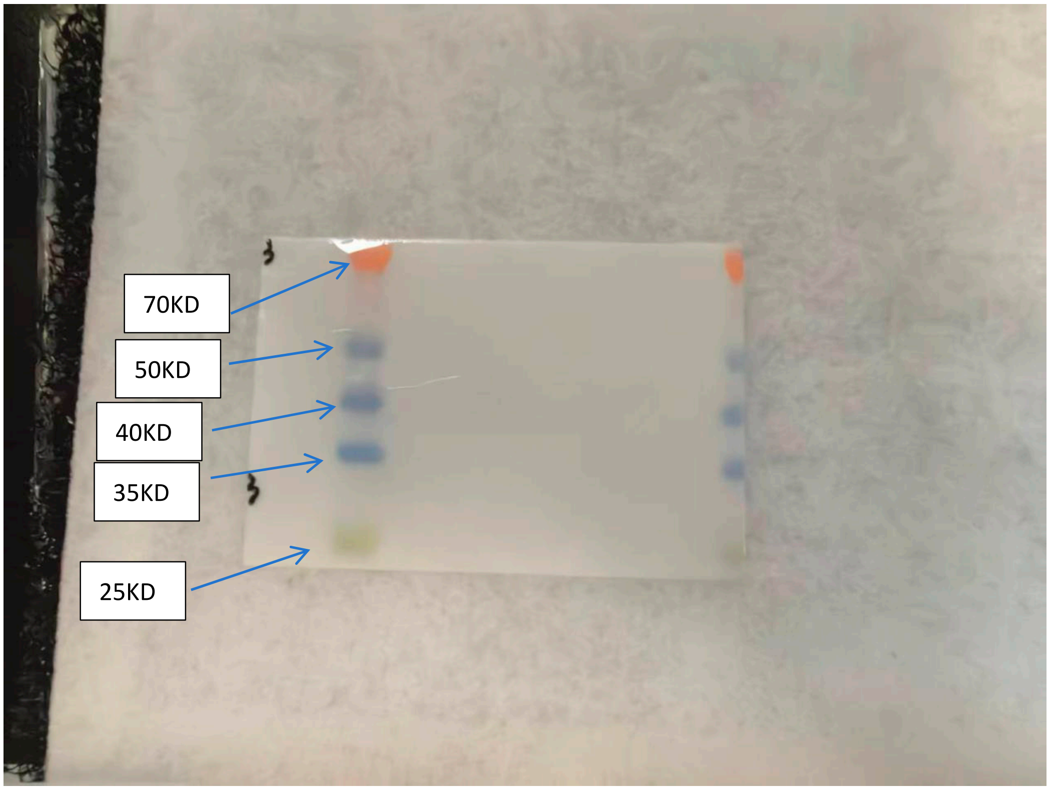
70KD
50KD
40KD
35KD
25KD

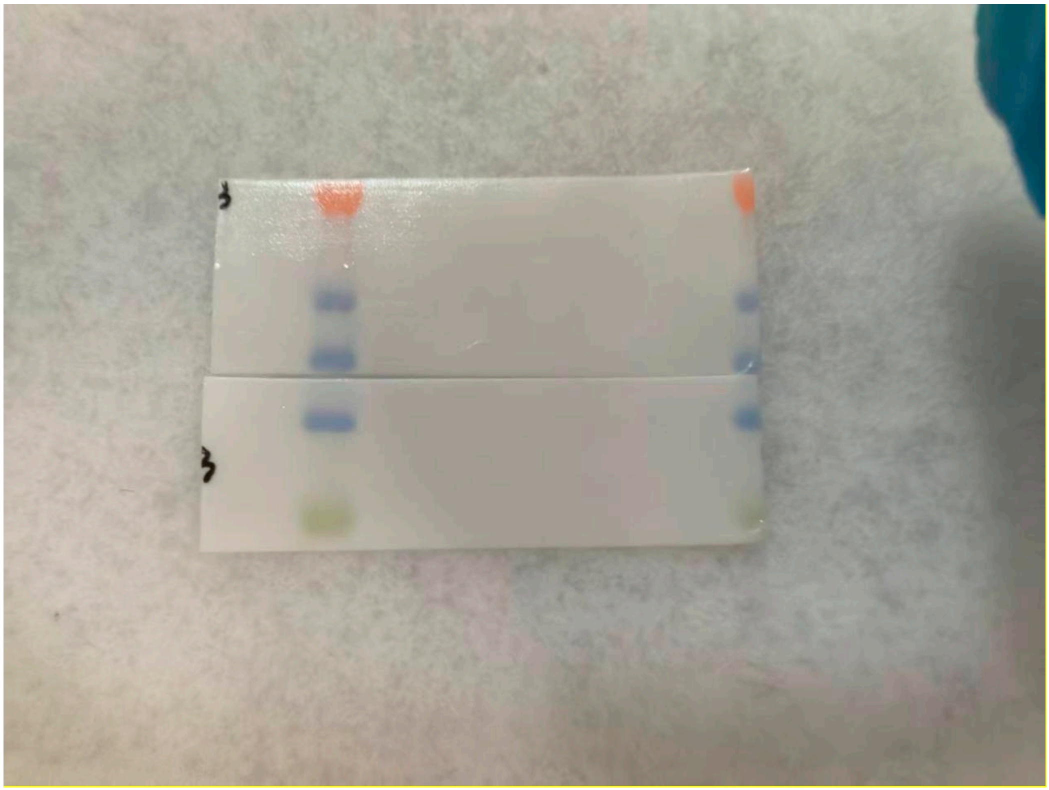

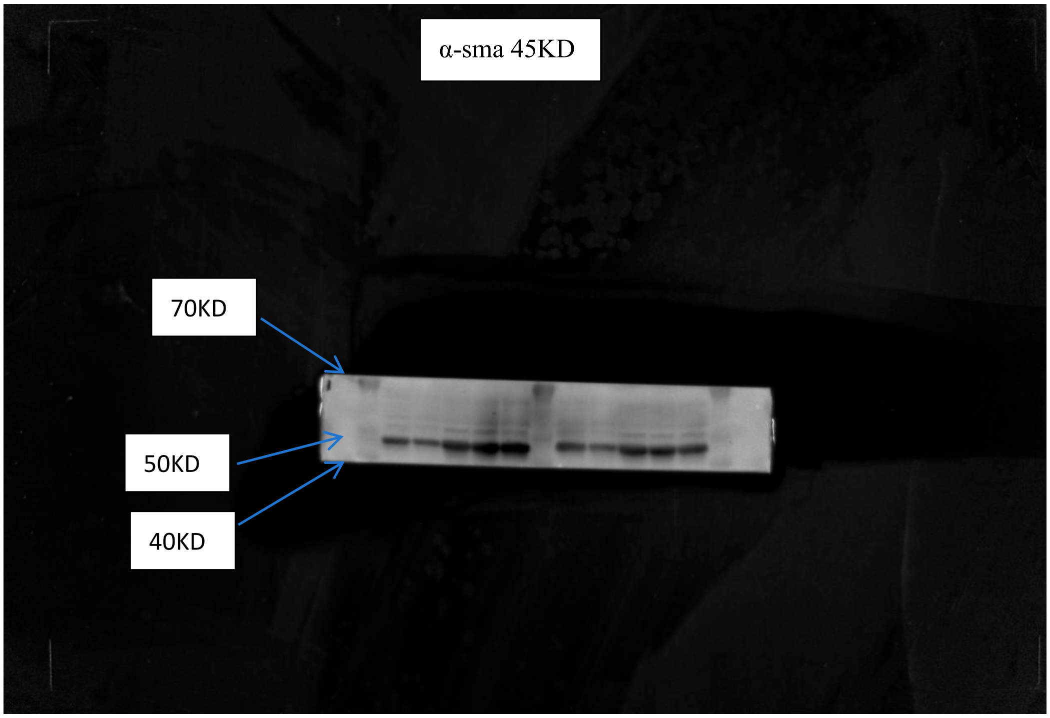
α-sma 45KD
70KD
50KD
40KD

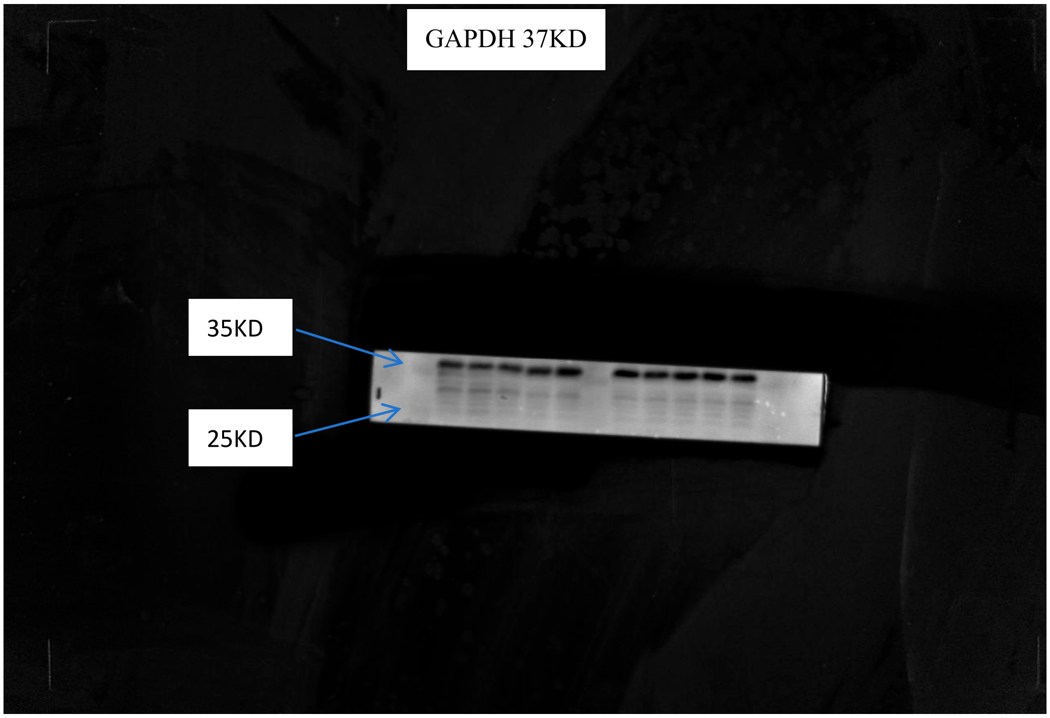
GAPDH 37KD
35KD
25KD

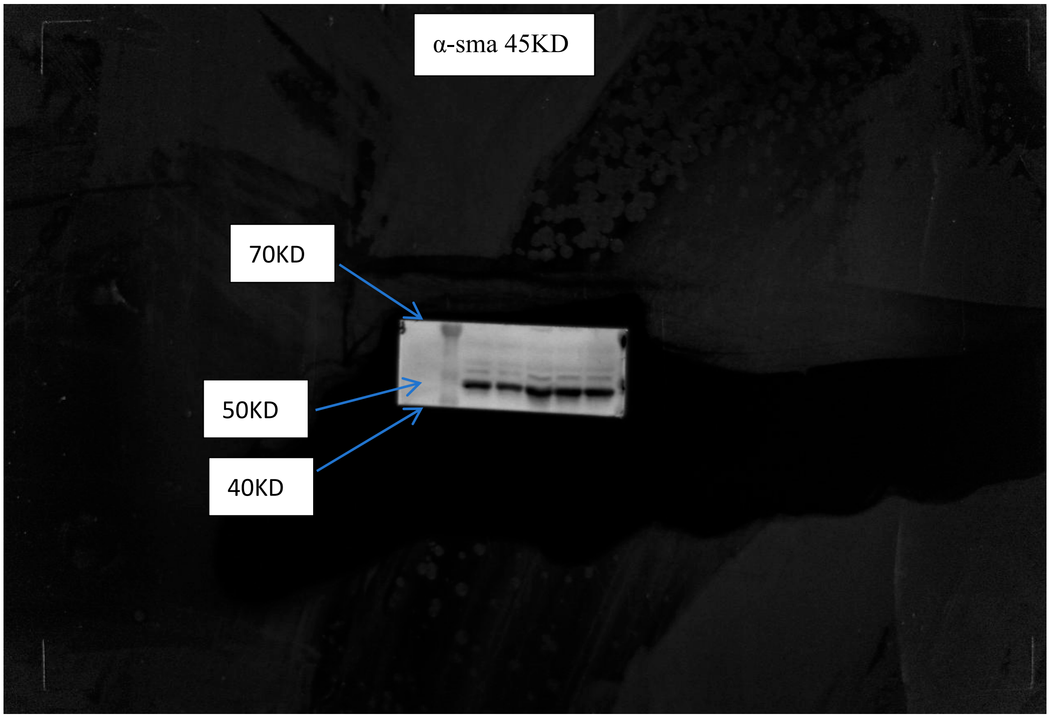
α-sma 45KD
70KD
50KD
40KD

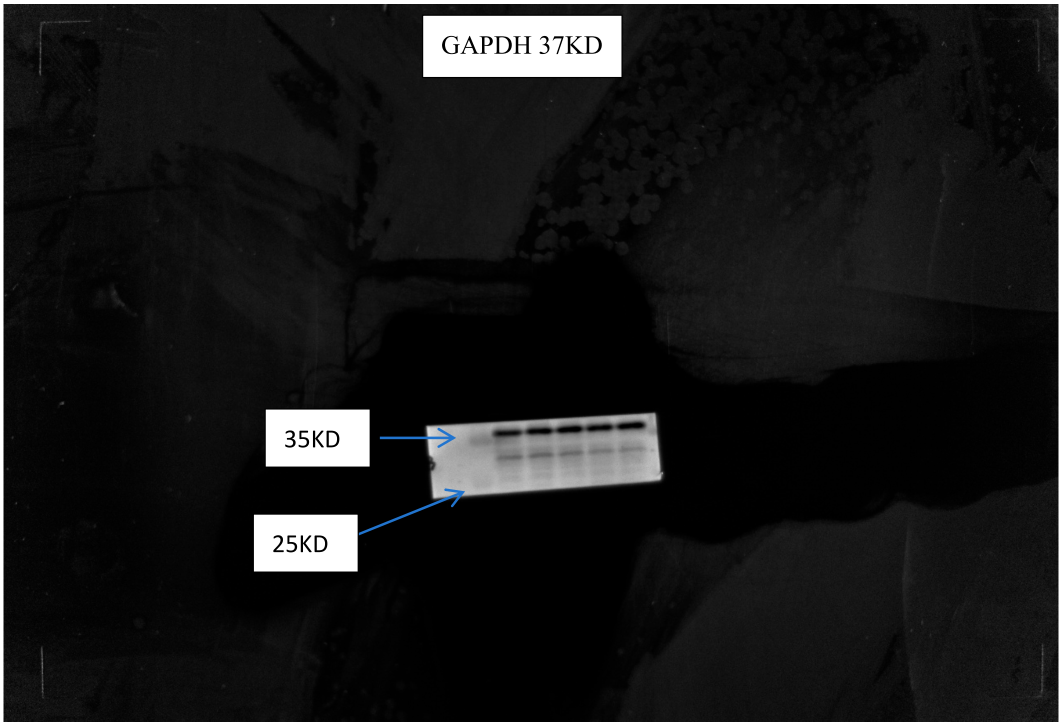
GAPDH 37KD
35KD
25KD

Supplementary Figures S21-S43

Figure S21–S43: Original uncropped and cropped Western blot images for Figure 8.

3 days

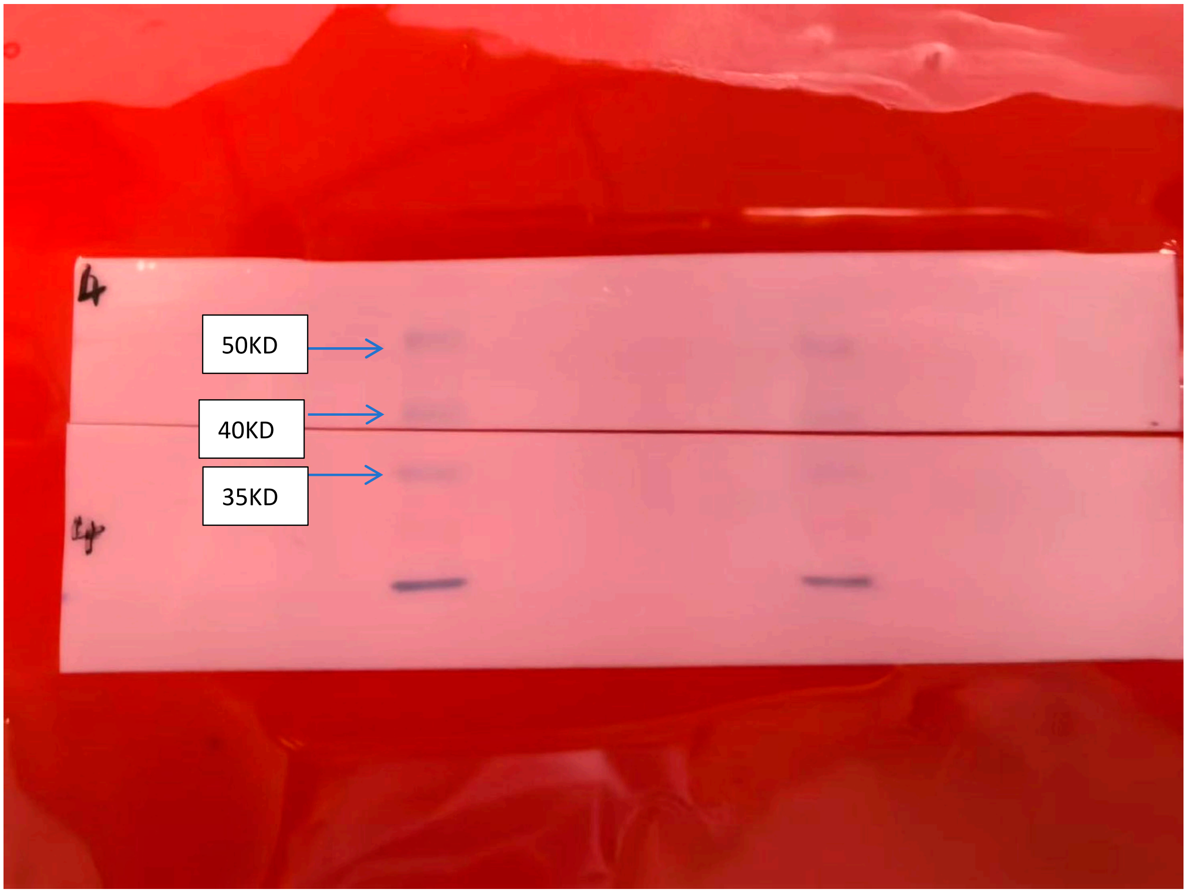

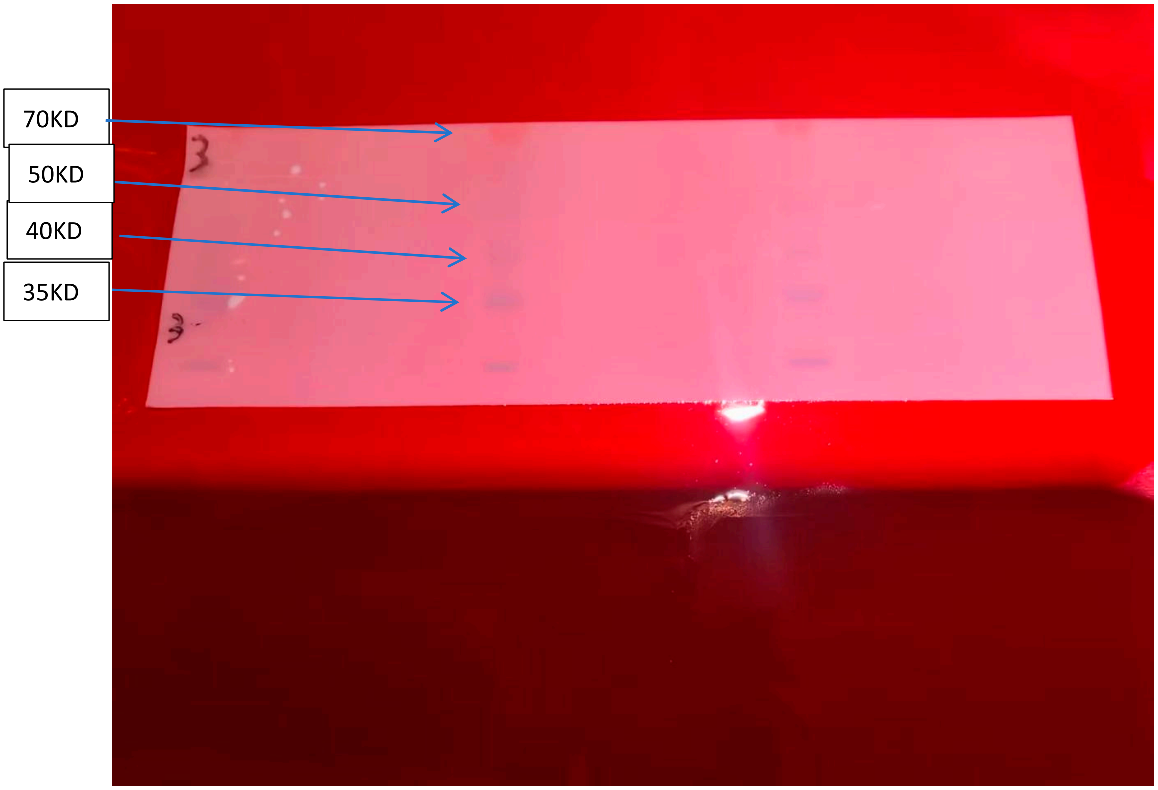
70KD
50KD
40KD
35KD

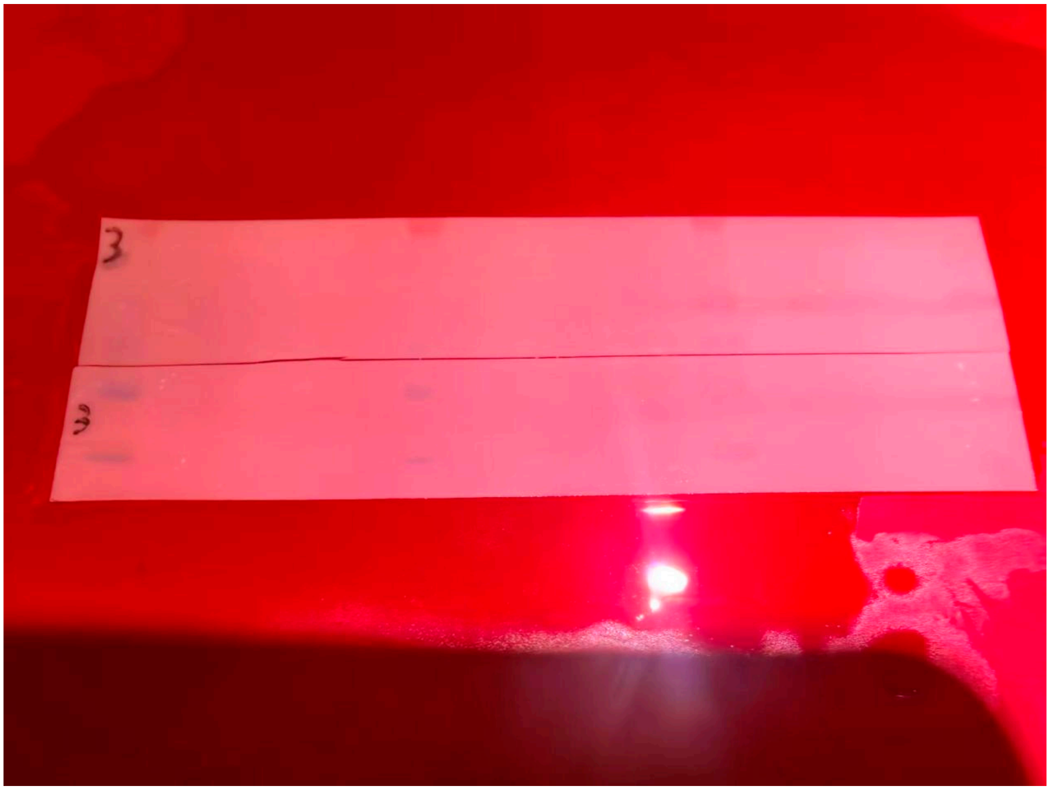

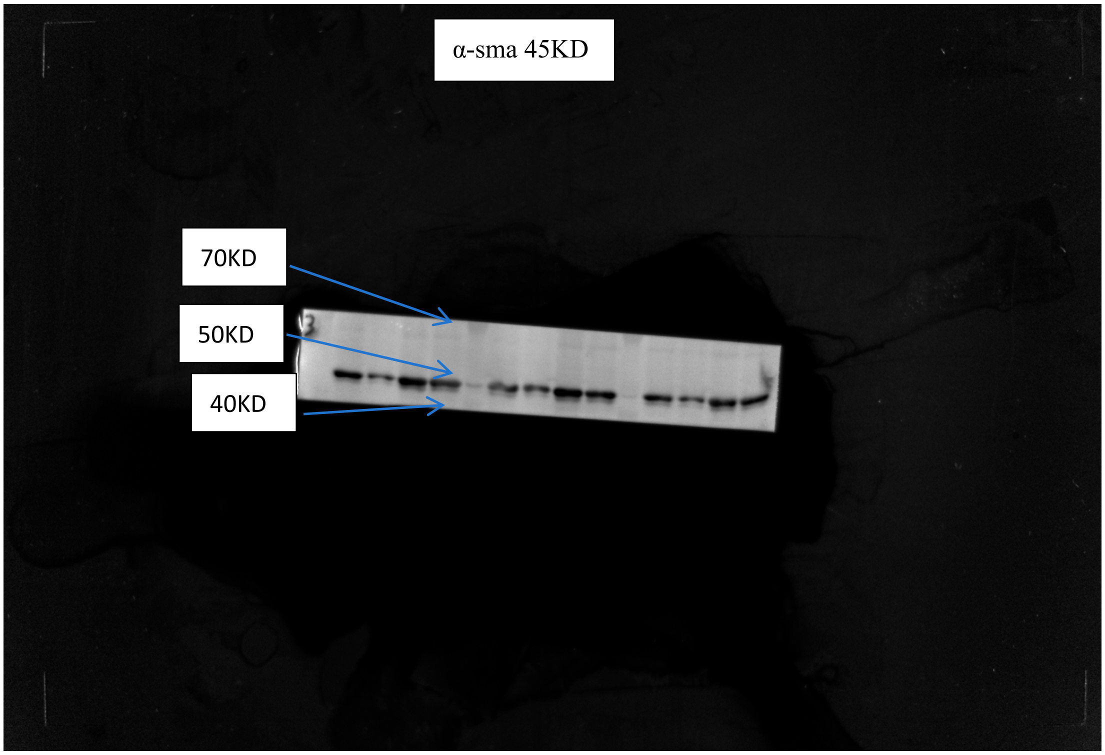
α-sma 45KD
70KD
50KD
40KD

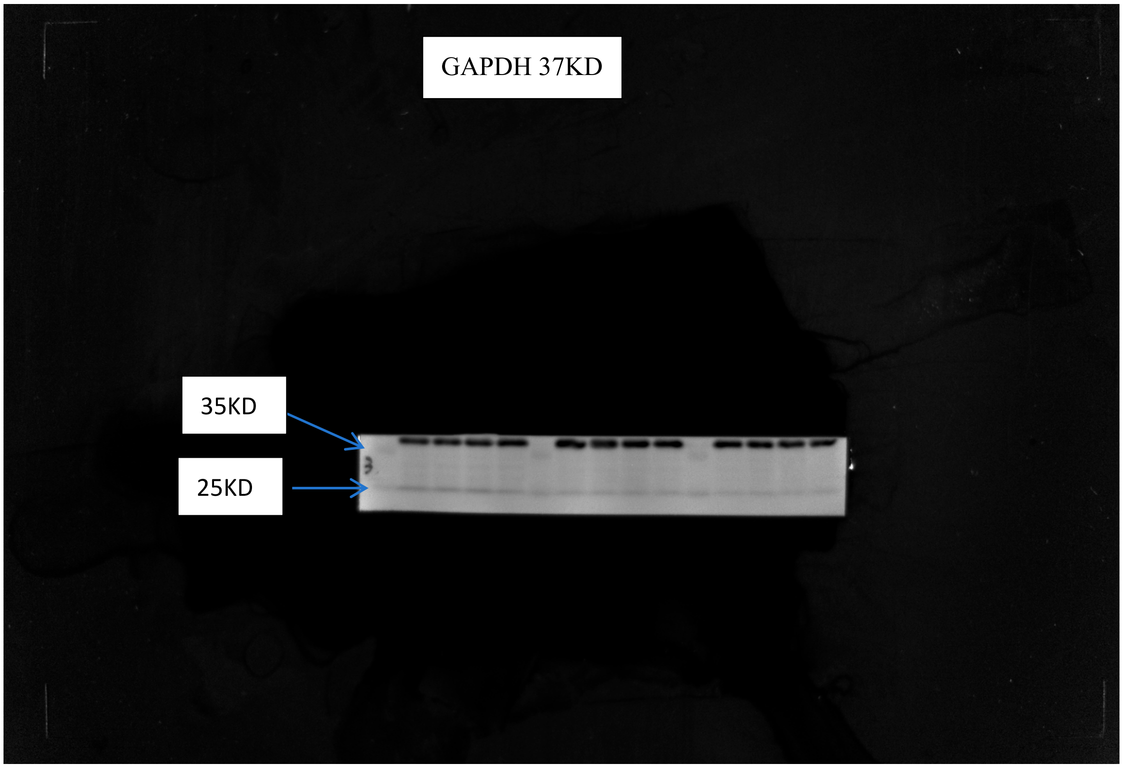
GAPDH 37KD
35KD
25KD

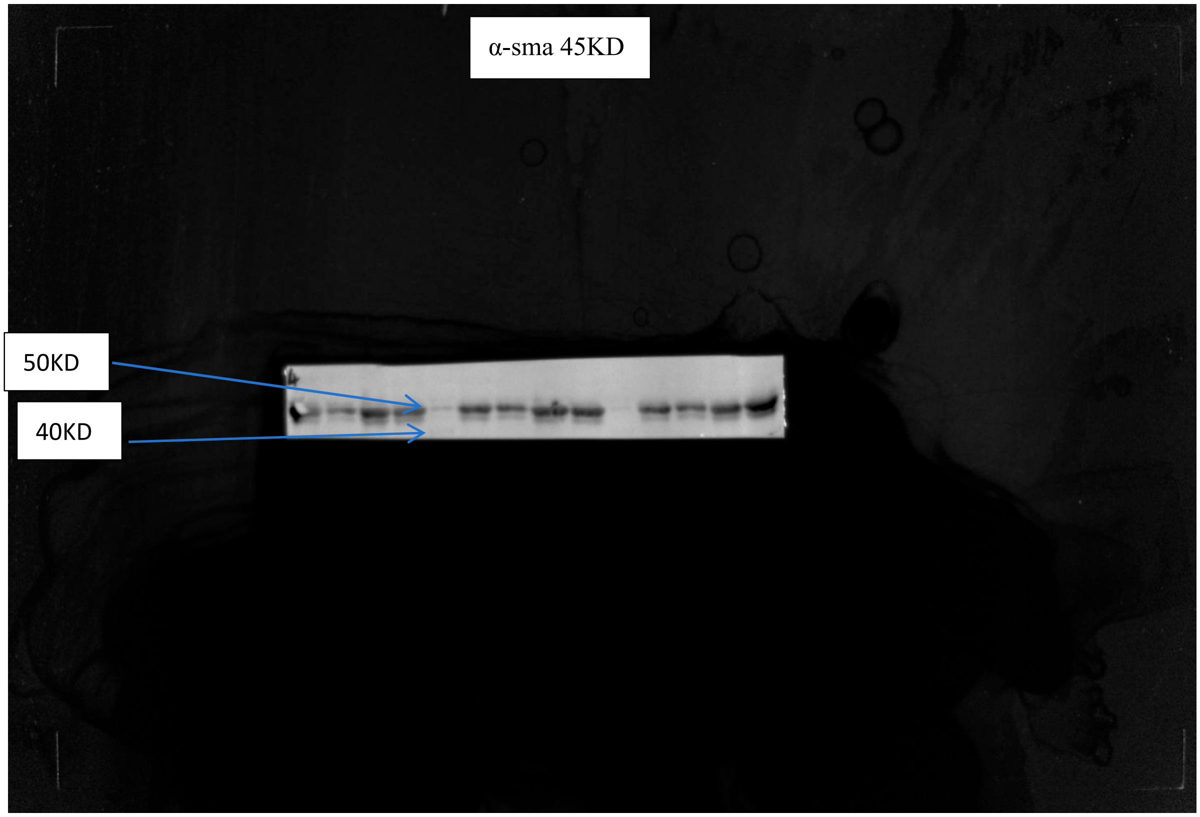
α-sma 45KD
50KD
40KD

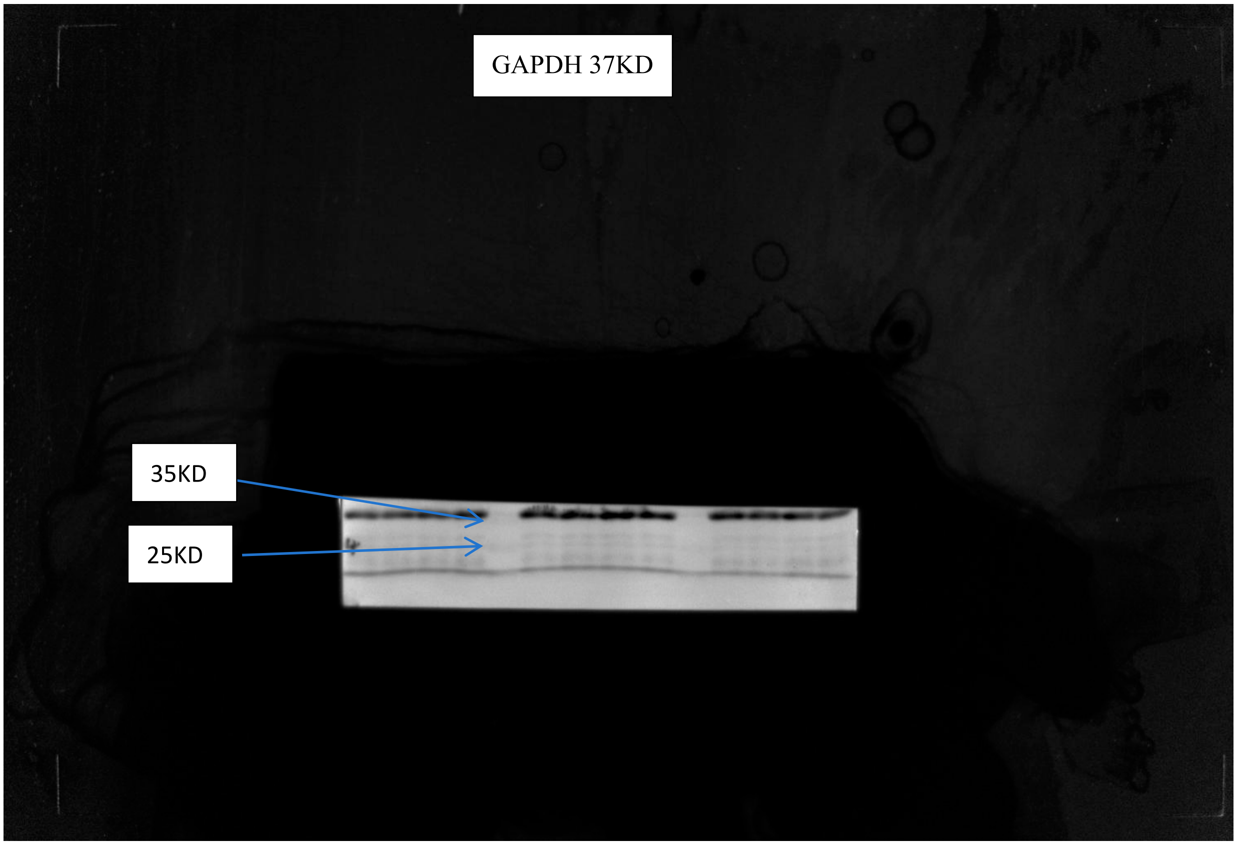
GAPDH 37KD
35KD
25KD

7 days

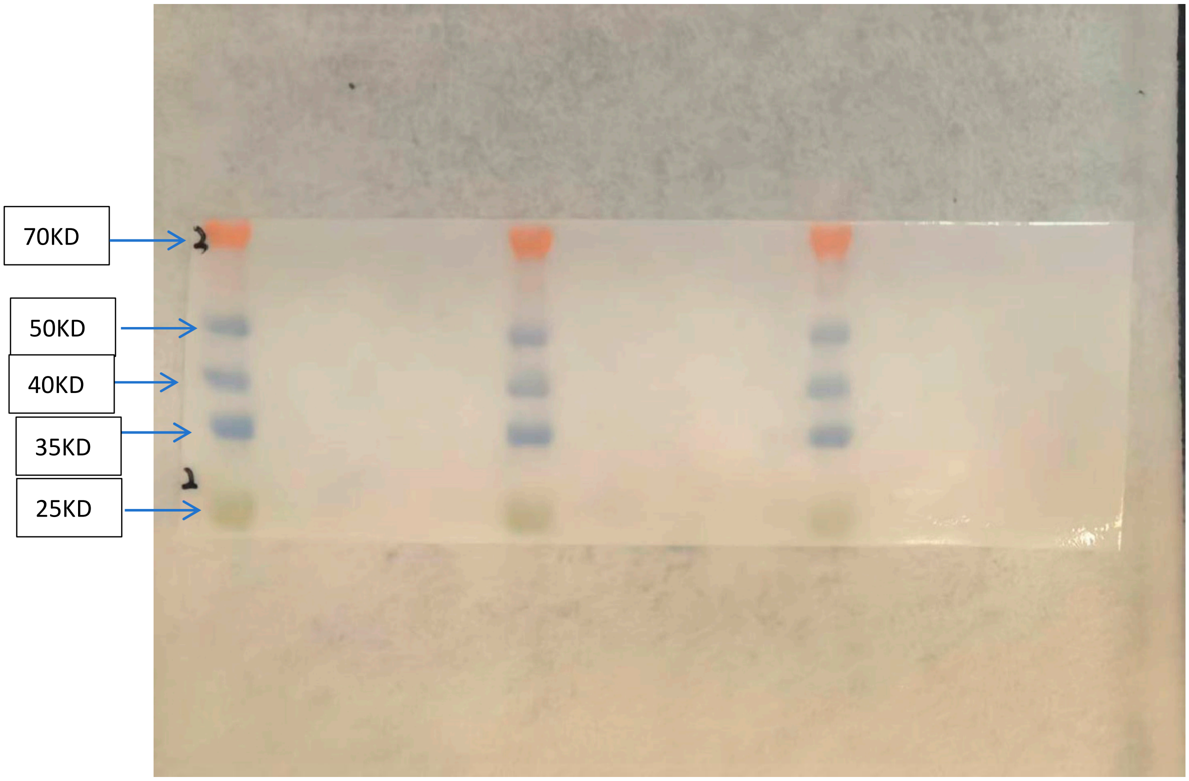

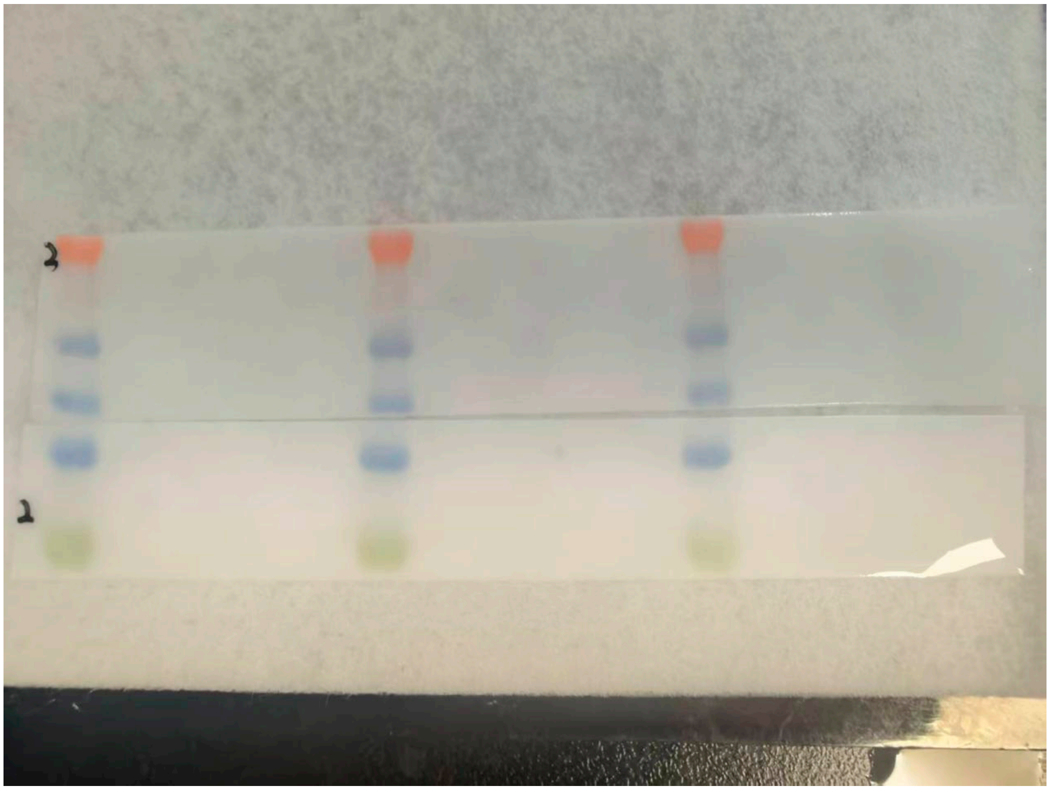

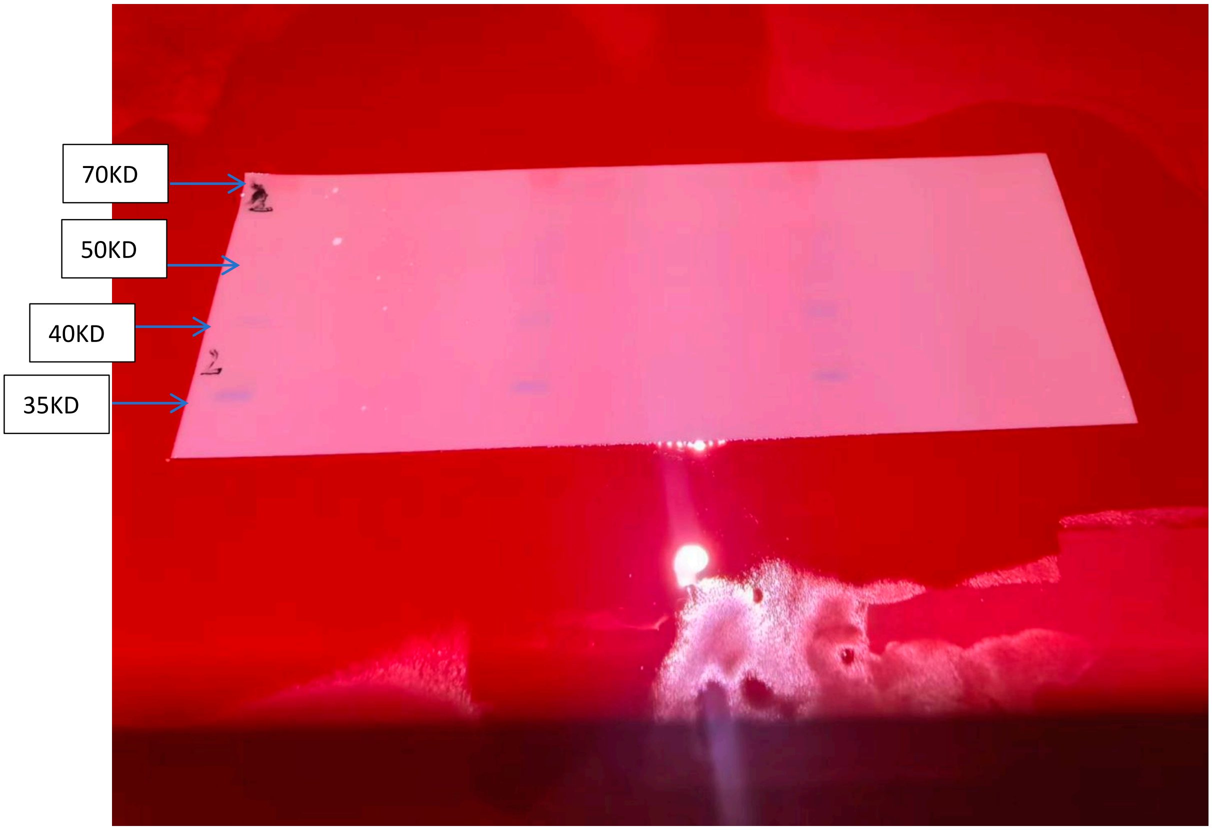
70KD
50KD
40KD
35KD

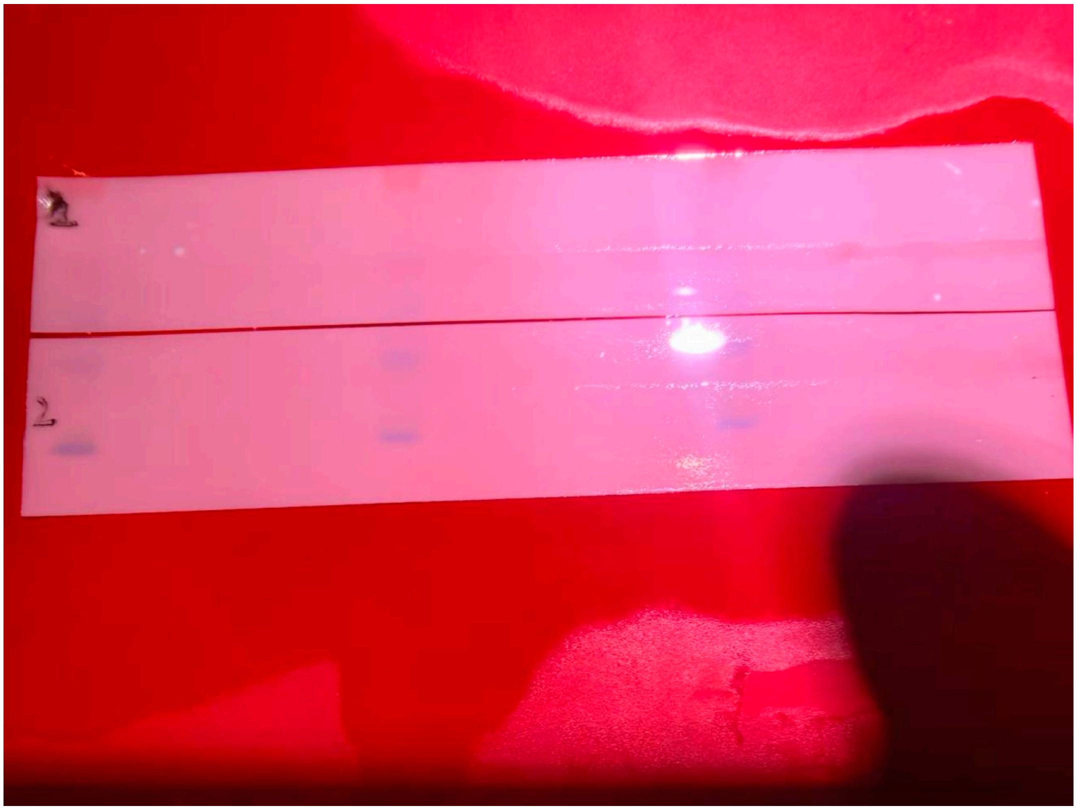

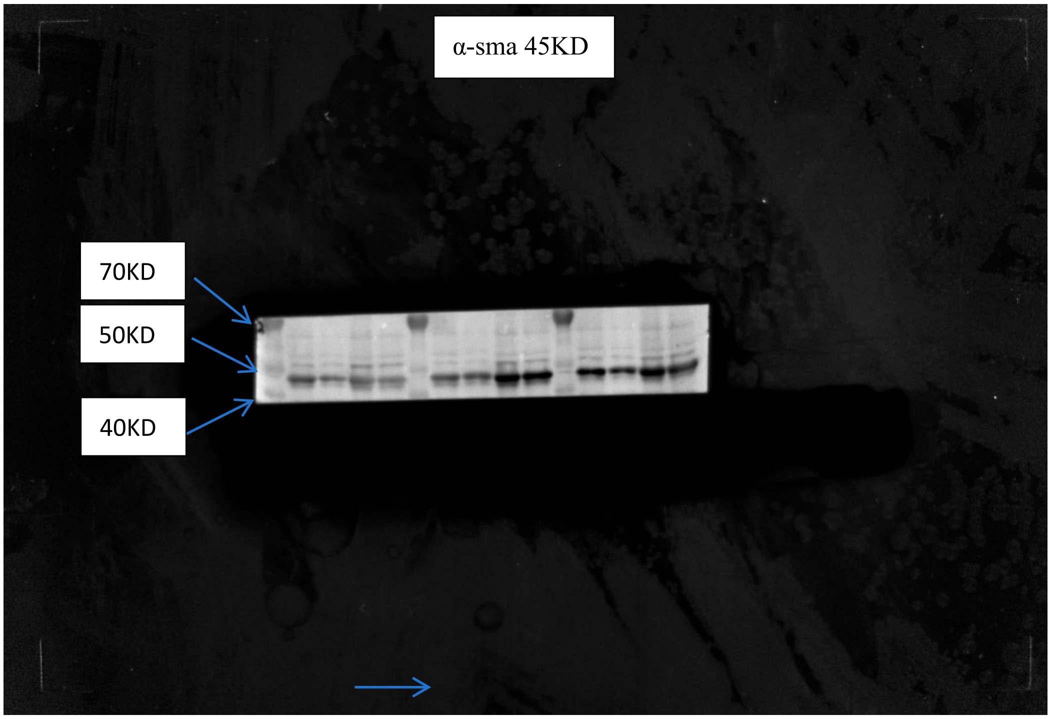
α-sma 45KD
70KD
50KD
40KD

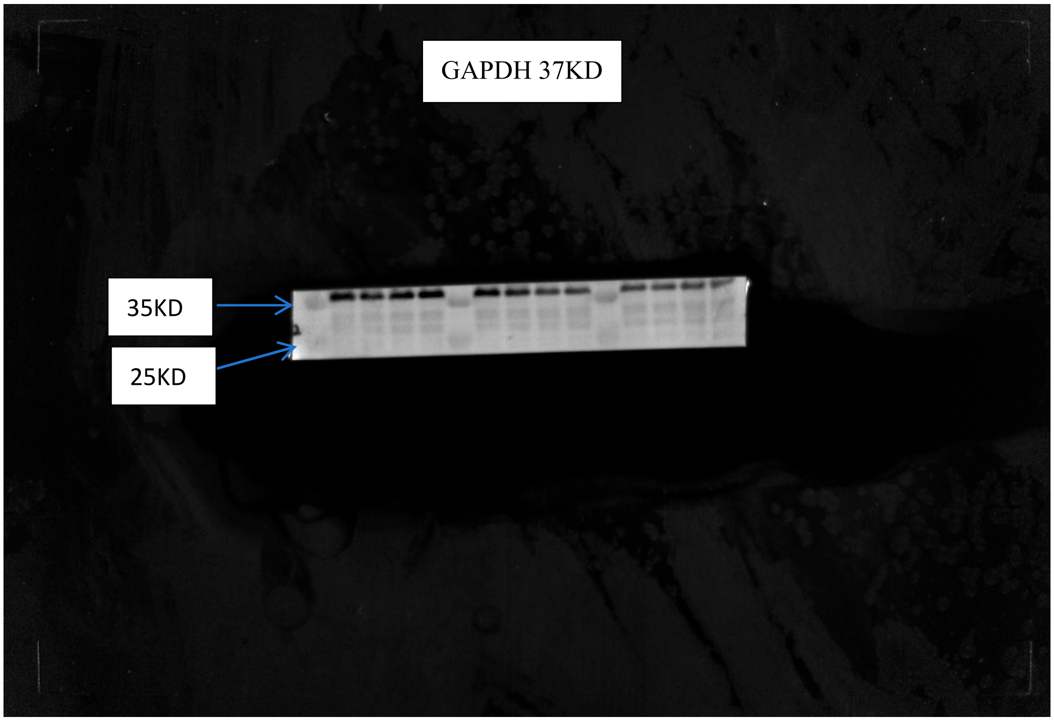
GAPDH 37KD
35KD
25KD

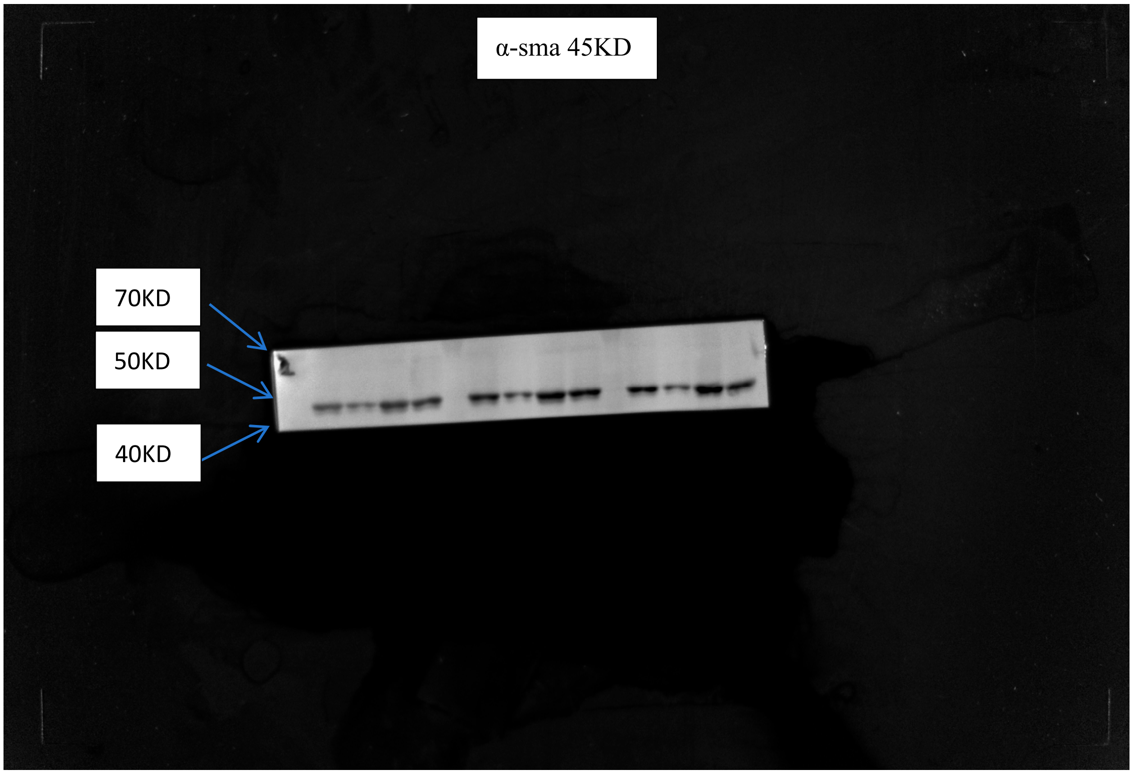
α-sma 45KD
70KD
50KD
40KD

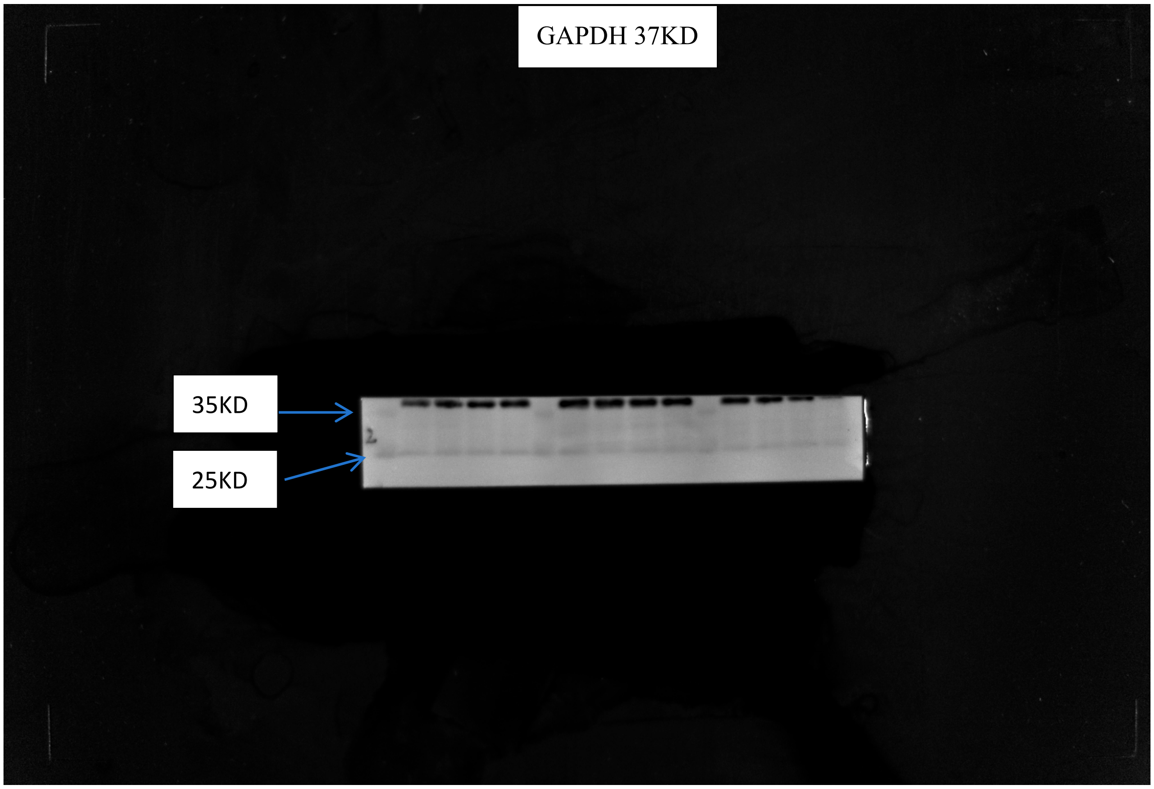
GAPDH 37KD
35KD
25KD

14 days

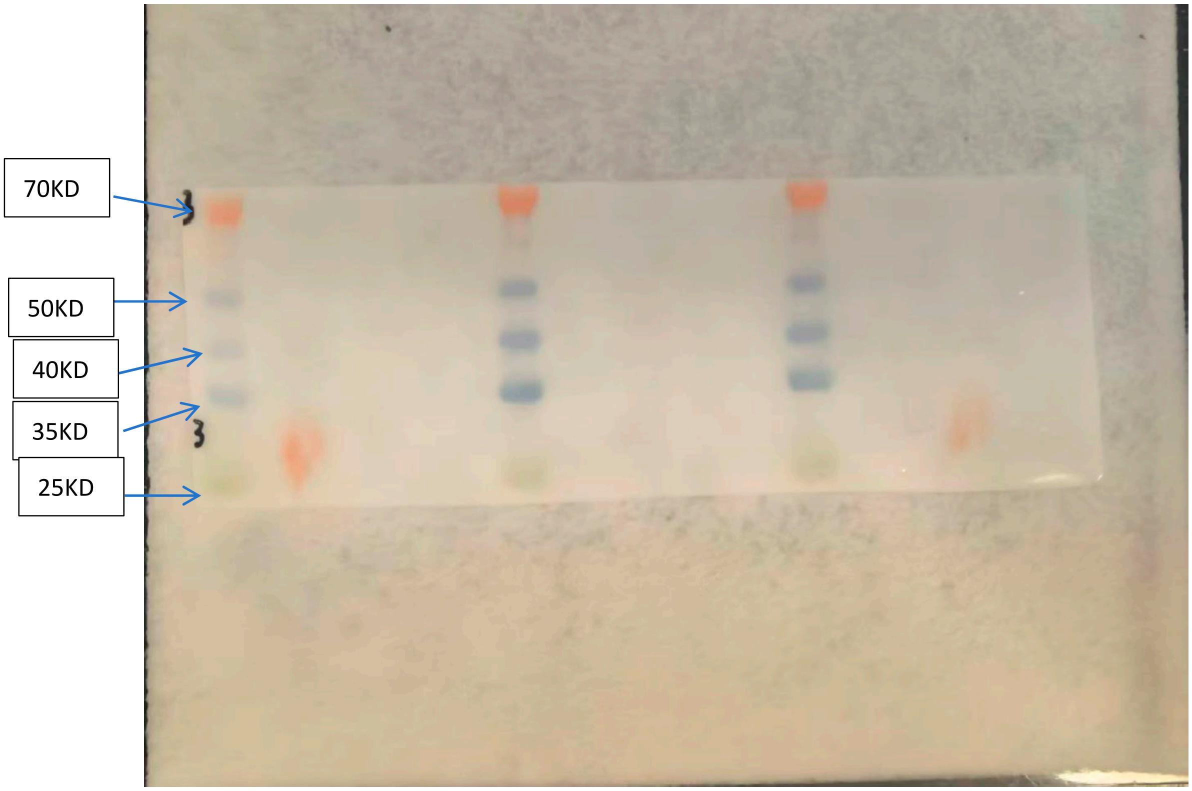

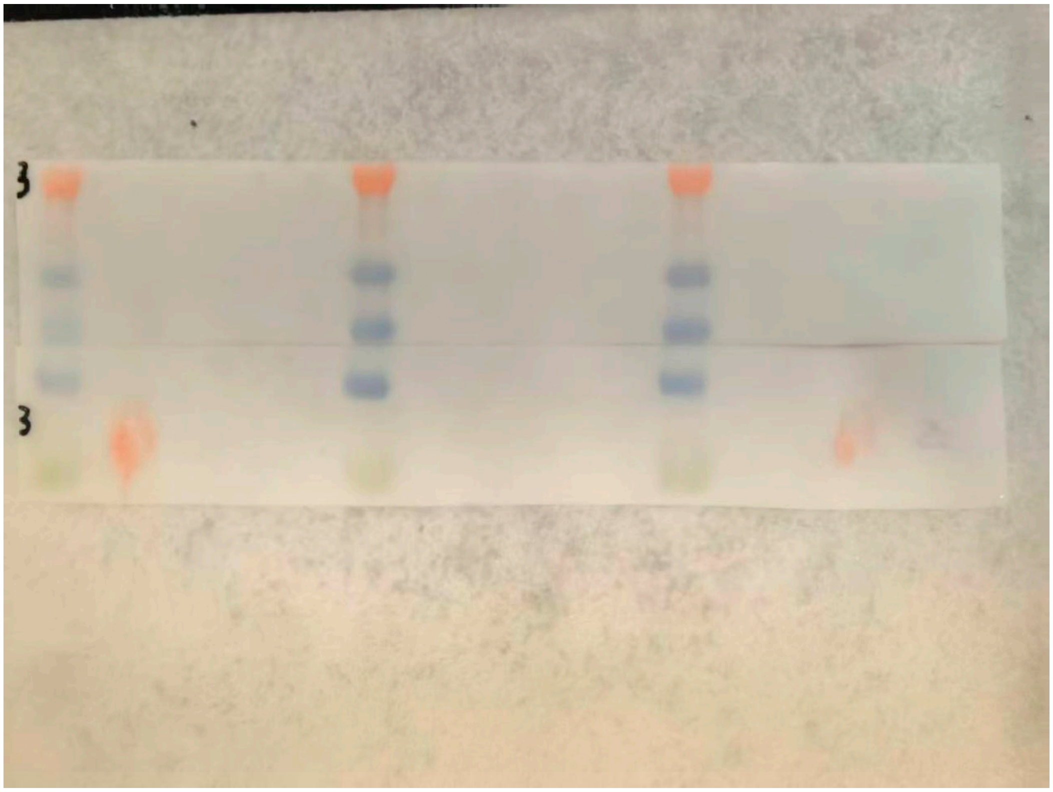

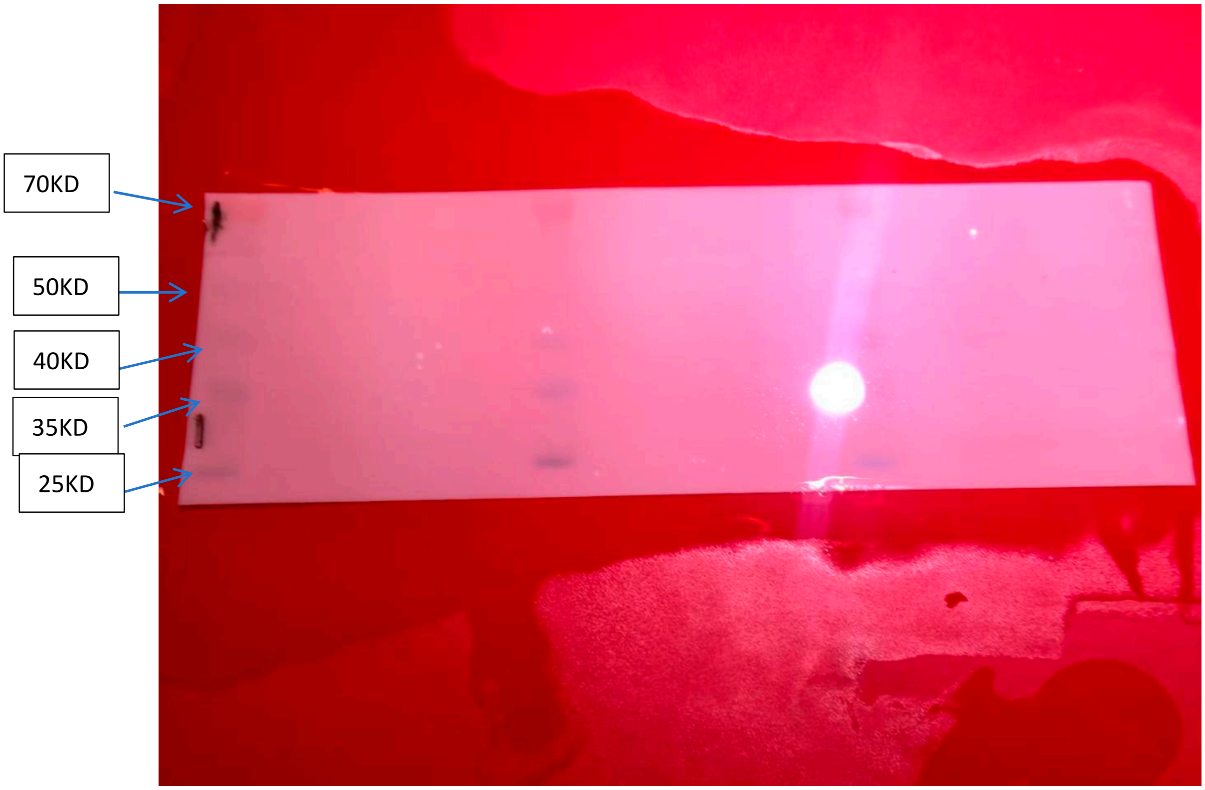
70KD
50KD
40KD
35KD
25KD

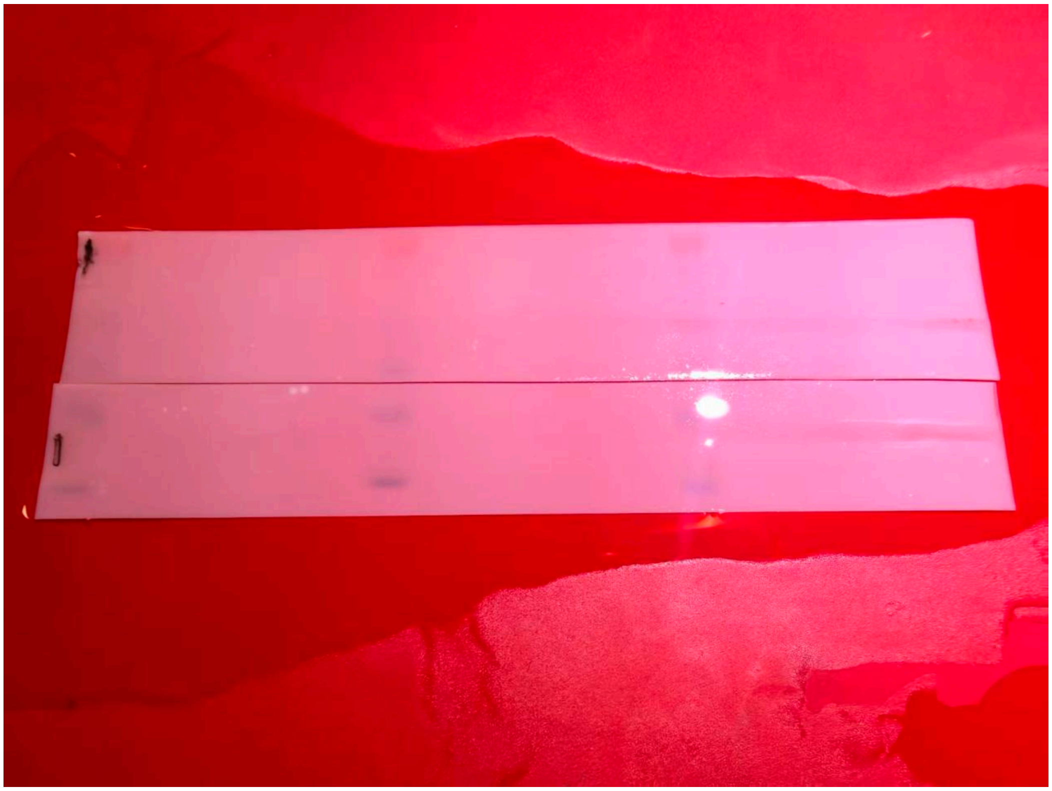

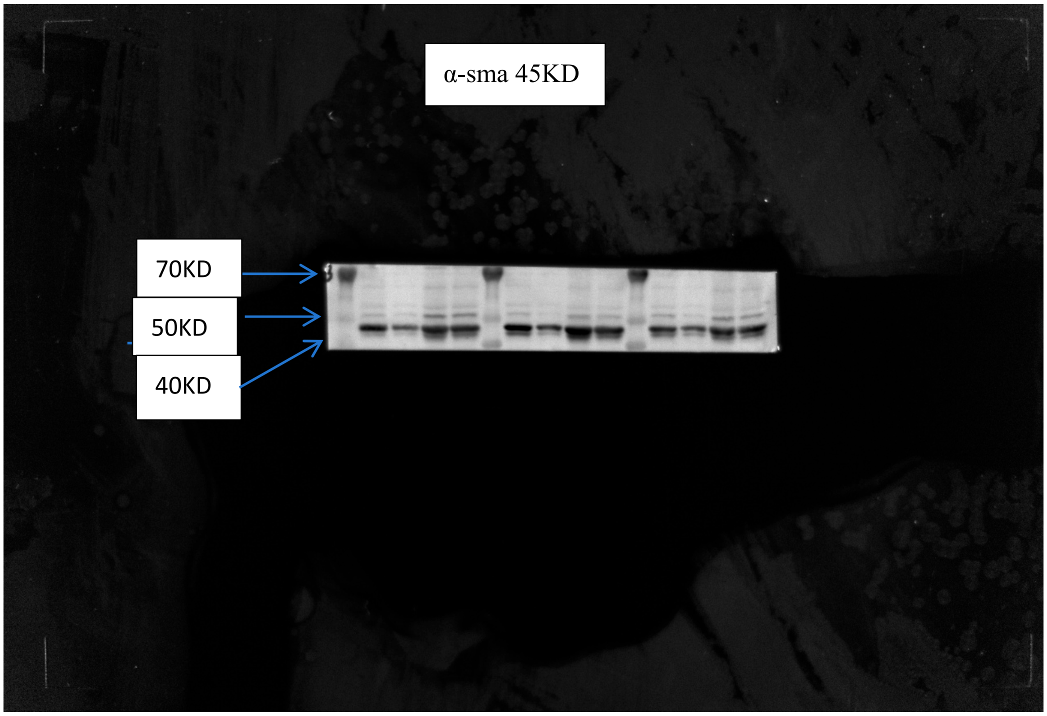
α-sma 45KD
70KD
50KD
40KD

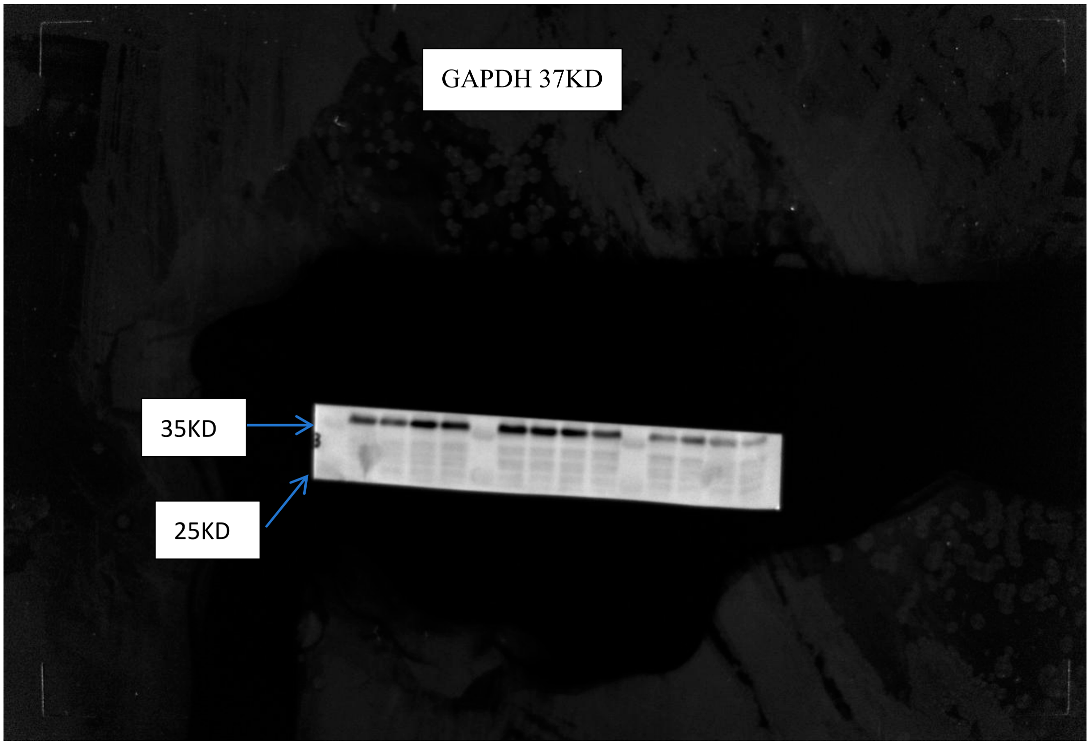
GAPDH 37KD
35KD
25KD

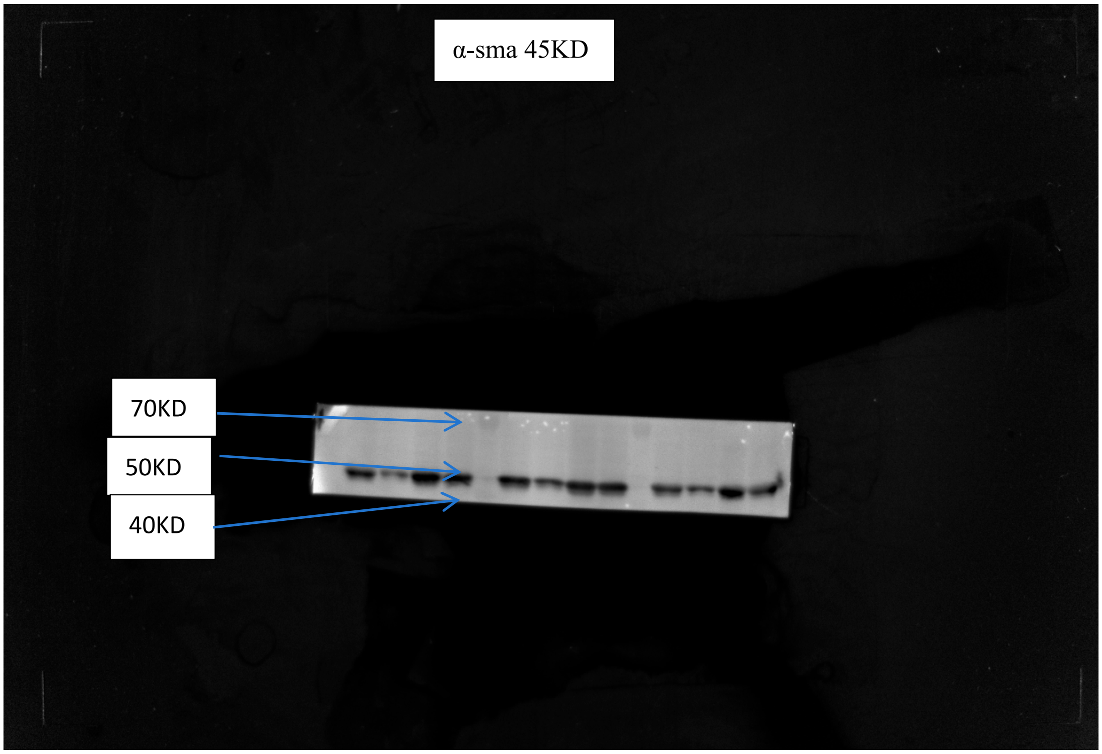
α-sma 45KD
70KD
50KD
40KD

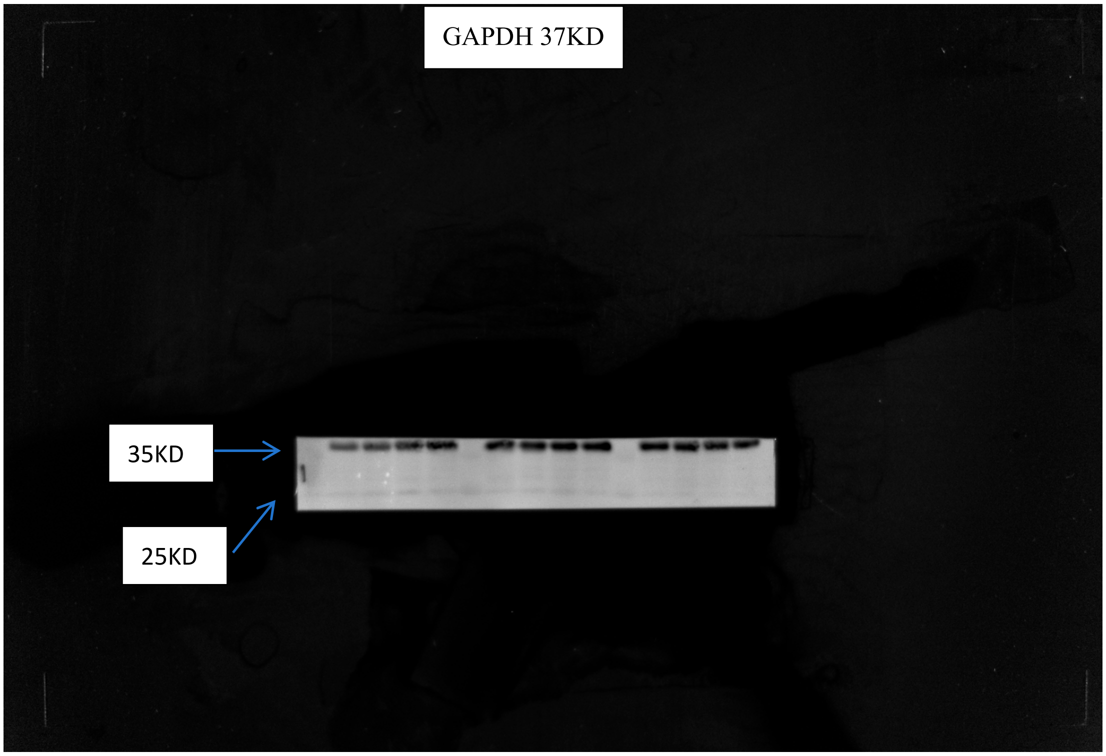
GAPDH 37KD
35KD
25KD
